# Identification of pyrazolopyridazinones as PDEδ inhibitors

**DOI:** 10.1038/ncomms11360

**Published:** 2016-04-20

**Authors:** Björn Papke, Sandip Murarka, Holger A Vogel, Pablo Martín-Gago, Marija Kovacevic, Dina C Truxius, Eyad K Fansa, Shehab Ismail, Gunther Zimmermann, Kaatje Heinelt, Carsten Schultz-Fademrecht, Alaa Al Saabi, Matthias Baumann, Peter Nussbaumer, Alfred Wittinghofer, Herbert Waldmann, Philippe I.H. Bastiaens

**Affiliations:** 1 Department of Systemic Cell Biology, Max Planck Institute of Molecular Physiology, D-44227 Dortmund, Germany; 2 Department of Chemical Biology, Max Planck Institute of Molecular Physiology, D-44227 Dortmund, Germany; 3 Structural Biology Group, Max Planck Institute for Molecular Physiology, D-44227 Dortmund, Germany; 4 Beatson Institute for Cancer Research, Bearsden, Glasgow G61 1BD, UK; 5 Lead Discovery Center GmbH, D-44227 Dortmund, Germany; 6 TU Dortmund, Faculty of Chemistry and Chemical Biology, D-44227 Dortmund, Germany

## Abstract

The prenyl-binding protein PDEδ is crucial for the plasma membrane localization of prenylated Ras. Recently, we have reported that the small-molecule Deltarasin binds to the prenyl-binding pocket of PDEδ, and impairs Ras enrichment at the plasma membrane, thereby affecting the proliferation of KRas-dependent human pancreatic ductal adenocarcinoma cell lines. Here, using structure-based compound design, we have now identified pyrazolopyridazinones as a novel, unrelated chemotype that binds to the prenyl-binding pocket of PDEδ with high affinity, thereby displacing prenylated Ras proteins in cells. Our results show that the new PDEδ inhibitor, named Deltazinone 1, is highly selective, exhibits less unspecific cytotoxicity than the previously reported Deltarasin and demonstrates a high correlation with the phenotypic effect of PDEδ knockdown in a set of human pancreatic cancer cell lines.

## Abstract (web summary)

PDEδ is a widely expressed factor that sustains the spatial organization and signalling of Ras family proteins. Here the authors describe the activity of Deltazinone 1, a new highly selective PDEδ inhibitor of KRAS-dependent cancer cell growth with low cytotoxic side effects.

## Graphical abstract

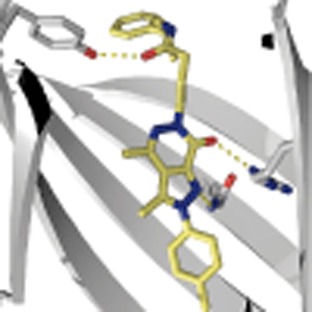

The products of the proto oncogene Ras in the GTP-bound state^1^ recruit effector proteins to the plasma membrane (PM) that activate proliferation- and survival-signalling in cells^2^,^3^. Oncogenic mutations of specific amino acids (AA), most commonly AA12, 13 and 61, maintain Ras in the constitutively active, GTP-bound state^4^, resulting in aberrant signalling. The most commonly mutated Ras isoform is KRas4B (from here on called KRas), which occurs in more than 90% of pancreatic, 45% of colorectal and 30% of lung tumours^4^.

Signal transduction of KRas strongly depends on its enrichment at the PM^5^. The localization motifs of KRas, a farnesylated cysteine and a polybasic stretch in the C-terminal hypervariable region^6^, are not sufficient to counter entropic equilibration to the extensive endomembrane surfaces^7^. A major role in counteracting this equilibration to endomembranes is played by the guanine nucleotide dissociation inhibitor (GDI)-like solubilization factor PDEδ^8^,^9^,^10^,^11^. It sequesters KRas from the cytosol by binding the farnesyl moiety, preventing that KRas binds to endomembranes and thereby enhancing its diffusion throughout the cell. KRas is then released in perinuclear membranes by the local activity of the release factor Arl2 (ref. 12), from where it is trapped by electrostatic interaction on the recycling endosome and shuttled back to the PM via vesicular transport^7^. Any interference with this cycle—such as competitive inhibition of PDEδ—will cause an entropy-driven relocalization of KRas to the extensive endomembrane surfaces^7^,^8^,^13^. Besides KRas, the PDEδ/Arl2 system is also crucial for maintaining membrane localization of other proteins of the Ras family, such as palmitoylated H- and N-Ras, as well as localization of the solely farnesylated Rheb on perinuclear membranes^5^. Delocalization of these Ras family molecules by interference with the PDEδ/Arl2 system is also expected to have a negative effect on cell growth and proliferation.

There have been many attempts to either target oncogenic Ras directly, its posttranslational modifications or downstream effectors with limited success^4^,^14^,^15^,^16^. Reducing PM localization of Ras through PDEδ inhibition raises alternative opportunities to impede oncogenic Ras signalling. The small-molecule Deltarasin affects the PM localization of KRas by competitively binding to the prenyl-binding pocket of PDEδ, relocating KRas to endomembranes. This KRas relocalization resulted in reduced proliferation of oncogenic KRas-dependent human pancreatic ductal adenocarcinoma cell lines (hPDACs)^13^.

However, subsequent detailed analysis of the dose–response curves characteristic for Deltarasin revealed that this PDEδ ligand displays a ‘switch-like' inhibition of proliferation; that is, the corresponding dose–response curve is very steep in the 3–8 μM range with a Hill coefficient of −5.3 to −10.8 (Supplementary Fig. 1). Such behaviour could arise from non-linear effects of Ras plasma membrane localization on signalling or could be indicative of general cytotoxicity by Deltarasin at high concentration and/or of interaction with additional target proteins in cells causing undesired side effects. Indeed, analysis of Deltarasin binding to additional proteins revealed that the compound also binds to different G-protein-coupled receptors, ion channels and transporters (Supplementary Table 1). Given this undesirable property of Deltarasin at concentrations >5 μM, validation of PDEδ as potential target for small-molecule interference with Ras localization and thereby also signalling activity, called for the development of a novel chemotype for inhibition of the Ras–PDEδ interaction, which would not display a comparable general cytotoxicity.

Herein, we describe the discovery of pyrazolopyridazinones as a novel PDEδ inhibitor chemotype that targets the prenyl-binding pocket of PDEδ with low nanomolar affinity. Structure-based ligand development led to the identification of the PDEδ ligand Deltazinone 1, which binds to PDEδ with high selectivity, shows anti-proliferative activity over a wide concentration range and is not generally cytotoxic. We demonstrate that inhibition of the PDEδ/Ras interaction by Deltazinone 1 is closely correlated to the phenotypic consequences of RNAi-mediated PDEδ knockdown in a panel of human pancreatic cancer cell lines.

## Results

### Synthesis of PDEδ inhibitors based on a different scaffold

**Figure 1:**
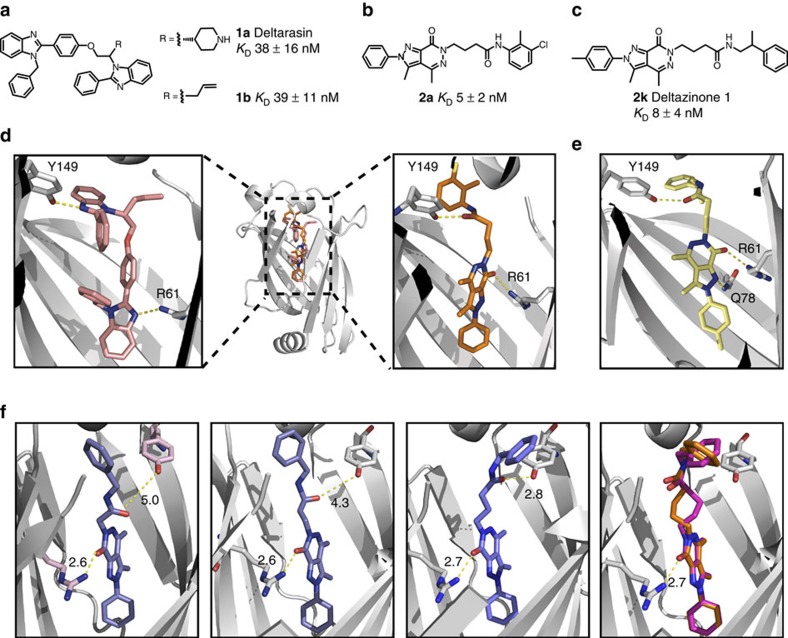
Identification of a novel KRas-PDEδ inhibitor chemotype. Structure and binding affinities (*K*_D_, determined by competitive fluorescence polarization assay of three independent experiments, see Supplementary Information) of (**a**) Deltarasin **1a** and allyl analogue **1b** (**b**) pyrazolopyridazinone derivative **2a** and (**c**) Deltazinone 1 **2k**. (**d**) Superimposition (middle) of the crystal structures of compounds **1b** (PDB code: 4JVB) and **2a** (PDB code: 5E80) in the PDEδ-binding pocket. The magnification on the left shows the structure of **1b** (1.75 Å) and of **2a** (2.60 Å) on the right side of the superimposition. Tyr149 (Y149) and Arg61 (R61) are shown as sticks to highlight the hydrogen bond interactions. (**e**) Predicted binding mode of Deltazinone 1, **2k** (best docking pose; Schrödinger, Maestro suite). Besides Y149 and R61, Gln78 (Q78) is also shown as stick to highlight the probable third H-bond interaction. (**f**) Best docking poses for a series of *N*-benzyl pyrazolopyridazinone derivatives containing linker chains with one, two, three (compound **2a**) and four methylene units. Distances for hypothetical hydrogen bonding with Tyr149 and Arg61 are shown.

To identify alternative chemotypes for the inhibition of the PDEδ/Ras interaction with potency similar to Deltarasin (Fig. 1a, *K*_D_=38±16 nM), an in-house library of ∼150,000 compounds, assembled considering structural diversity of the underlying scaffolds, drug-likeness, coverage of different established drug target classes and inspiration by natural products, was subjected to high-throughput screening by means of the previously developed AlphaScreen Technology^13^. The screen revealed pyrazolopyridazinone **2a** (*K*_D_=5±2 nM) as ligand of the prenyl-binding pocket of PDEδ (Fig. 1b).

Structure-based development of the pyrazolopyridazinone hit class was enabled by a crystal structure of inhibitor **2a** in complex with PDEδ at a resolution of 2.6 Å (Fig. 1d) which revealed that, like inhibitors of the Deltarasin-type, heterocycle **2a** employs H-bonds to Tyr 149 and Arg 61 for binding to PDEδ^13^. Initial screening and molecular modelling investigations (Schrödinger, Maestro suite) revealed that a C-3 linker between the two hydrogen-bonding groups is important and that shortening of the linker would abrogate H-bond formation between the amide carbonyl and Tyr 149 (Fig. 1f). Therefore, a C-3 linker was kept for further inhibitor development.

Since anilides display low metabolic stability, we searched for an alternative to the anilide embodied in hit compound **2a**. Initial investigations and modelling revealed benzyl- and homobenzylamides as potential inhibitors, since they could adopt a conformation in which an H-bond with Tyr 149 is formed and the empty space in the PDEδ pocket is filled. Therefore, a series of compounds with these constraints was synthesized and investigated (Table 1). As shown in Table 1, benzyl amide **2c** also binds to PDEδ with nanomolar affinity. Reduction of the ring size (entry 2) or incorporation of a substituent on the phenyl ring was not favourable (entries 4 and 5). In addition, modelling indicated that a small substituent might be attached to the *N*-phenyl moiety, pointing deep into the farnesyl-binding site. Indeed, incorporation of a *p*-methyl substituent into the pyrazole *N*-phenyl ring resulted in low nanomolar affinity (entries 2, 3, 6 versus 7, 8, 9). Besides benzyl amides, homobenzyl amides are also very potent inhibitors displaying low nanomolar affinities (entries 6, 9–11).

Compound **2k** (Fig. 1c) was selected because it embodies one metabolically unstable benzylic position less than compound **2j**. As compared with compound **2i,** we assumed that the presence of an additional methyl group at the benzylic position in **2k** would further stabilize the compound with respect to metabolic degradation. Based on the fact that pyrazolopyridazinone **2a** binds to PDEδ like the Deltarasin-type inhibitors (Fig. 1d), molecular modelling experiments indicated that **2k** will form the same hydrogen bonds as Deltarasin and may additionally form a hydrogen bond with glutamine 78 (Fig. 1e). Compound **2k** was, therefore, subjected to a panel of biochemical and pharmacological screens to determine its selectivity profile and possible interactions with other pharmacologically relevant proteins. This compound proved to be more selective as compared with Deltarasin in the selected panel of targets (Supplementary Fig. 2 and Supplementary Table 1) and was chosen for further investigations in cells. We named this compound Deltazinone 1.

### Deltazinone 1 inhibits the PDEδ–Ras interaction

**Figure 2:**
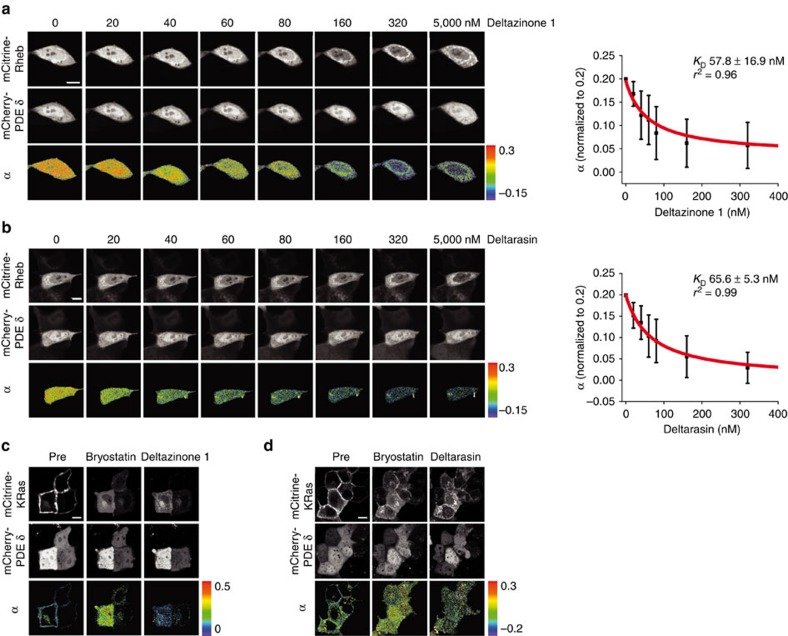
In-cell measurement of the effect of Deltarasin or Deltazinone 1 on the interaction between Ras proteins and PDEδ. Dose–response measured by the interacting fraction (*α*) of mCitrine-Rheb with mCherry-PDEδ for Deltazinone 1 (**a**) and Deltarasin (**b**) as determined by FLIM-FRET. The inhibitor concentration is indicated at the top of each image in nM. Right panels, fit of averaged dose–response±s.e.m. of nine independent experiments to a binding model (see Methods) yielded an in-cell *K*_D_ of 58±17 nM for Deltazinone 1 (**a**) and 66±5 nM for Deltarasin (**b**). Interacting fraction (*α*) of mCitrine-KRas with mCherry-PDEδ on 5 min treatment with 1 μM Bryostatin-1 and subsequent addition of 5 μM Deltazinone 1 (**c**) or 5 μM Deltarasin after 5 min (**d**). First row: fluorescence intensity distribution of mCitrine-Rheb (**a**,**b**) or mCitrine-KRas (**c**,**d**); second row: fluorescence intensity distribution of mCherry-PDEδ; third row: molar fraction *α* (false colour coded) of interacting mCitrine-Rheb with mCherry-PDEδ (**a**,**b**) or mCitrine-KRas with mCherry-PDEδ (**c**,**d**). Scale bars, 10 μm.

To compare the inhibition of PDEδ by Deltazinone 1 to Deltarasin, the interaction between farnesylated Ras proteins and PDEδ was measured in live cells by fluorescence lifetime imaging microscopy of Förster resonance energy transfer (FLIM-FRET)^13^. For this, Madin–Darby canine kidney (MDCK) cells were transiently transfected with Rheb N-terminally fused to a yellow fluorescent protein (mCitrine-Rheb), and PDEδ fused to a red fluorescent protein variant (mCherry-PDEδ). Rheb is a farnesylated Ras protein that lacks the polybasic stretch in the hypervariable region, resulting in a low membrane affinity and an enhanced fraction that can interact with PDEδ in the cytoplasm. This facilitates the detection of small-molecule effects on the interaction between farnesylated proteins and PDEδ by FLIM-FRET. The fluorescence patterns of mCitrine-Rheb and mCherry-PDEδ in the absence of the inhibitor showed a homogeneous distribution, demonstrating that mCitrine-Rheb is solubilized by mCherry-PDEδ (Fig. 2a,b). This was reflected in the high molar fraction (α) of interacting Rheb-PDEδ as measured by FLIM-FRET. Increasing concentrations of Deltazinone 1 or Deltarasin resulted in a reduced interaction of mCitrine-Rheb with mCherry-PDEδ that was paralleled by a relocalization of mCitrine-Rheb to endomembranes. Dose-dependent measurements yielded an ‘in-cell' *K*_D_ of 58±17 nM for Deltazinone 1 and 66±5 nM for Deltarasin.

To measure whether both inhibitors also affect the interaction of KRas with PDEδ, MDCK cells were transiently transfected with mCitrine-KRas and mCherry-PDEδ. The cytosolic fraction of KRas is much lower than the one of Rheb due to its higher PM affinity caused by its polybasic stretch^5^. This leads to a smaller fraction of mCitrine-KRas interacting with mCherry-PDEδ at steady state in the cytoplasm, making the interaction difficult to detect with FLIM-FRET. To increase the fraction of KRas that interacts with PDEδ, the Protein Kinase C (PKC) activator Bryostatin, which induces serine (S181) phosphorylation on KRas^17^, was applied, resulting in a reduced electrostatic interaction with the PM and increased cytosolic pool of mCitrine-KRas. Subsequent addition of 5 μM Deltazinone 1 (Fig. 2c) or Deltarasin (Fig. 2d) abolished the interaction between mCitrine-KRas and mCherry-PDEδ and resulted in relocalization of KRas to endomembranes.

### Deltazinone 1 impairs growth of KRas-dependent hPDAC cells

**Figure 3:**
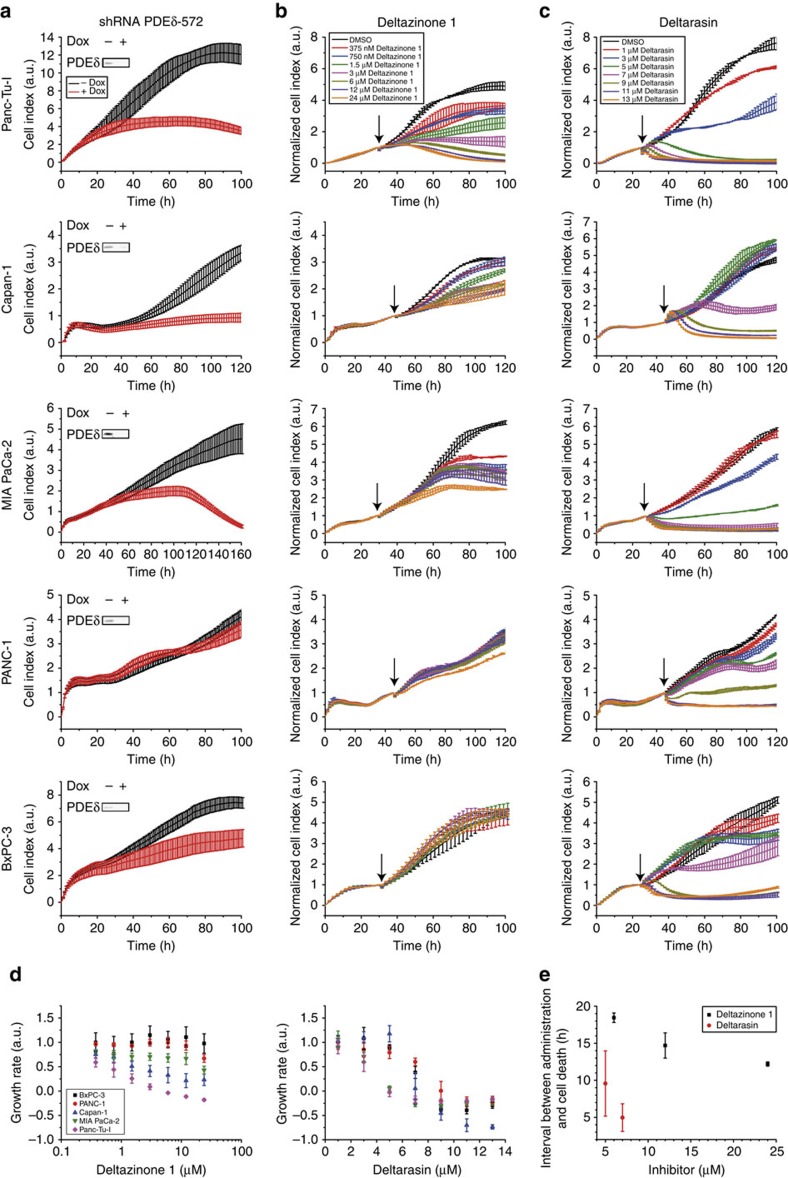
Comparison of the RTCA growth profiles of pancreatic cancer cell lines after PDEδ knockdown to PDEδ inhibition by Deltazinone 1 or Deltarasin. The first column (**a**) shows the effects of doxycycline-induced PDEδ knockdown on cell proliferation. Cell indices were measured in the presence (red) or absence (black) of doxycycline. Doxycycline was added at the beginning of the measurement. The inset in each profile displays the respective PDEδ protein levels determined by western blot in absence and presence of doxycycline after 72 h. The second (**b**) and third column (**c**) show dose-dependent effects of Deltazinone 1 and Deltarasin on cell proliferation, respectively. The small-molecule PDEδ inhibitors were added at the indicated time points (arrow) and concentrations. The cell indices were normalized to the time of administration. Cell indices±s.d. were measured in duplicates (Capan-1, MIA PaCa-2 and BxPC-3) or triplicates (Panc-Tu-I and PANC-1). (**d**) Deltazinone 1 or Deltarasin dose versus growth-rate±s.d. as determined by the integrated area below the curve in the first 60 h after drug administration, normalized to the DMSO control. (**e**) Time interval between PDEδ inhibitor administration and observed cell death in Panc-Tu-I cells. The mean intervals±s.d. are depicted for each drug.

To compare the effects of PDEδ knockdown with small-molecule PDEδ inhibition on proliferation, impedance-based real-time cell analyser (RTCA) measurements were performed in a panel of pancreatic cancer cell lines (Table 2, Fig. 3). For this, all pancreatic cancer cell lines were transduced with the previously reported doxycycline-induced short hairpin RNA (shRNA) construct against PDEδ^13^.

The doxycycline-induced PDEδ knockdown in the oncogenic KRas-dependent human carcinoma cell lines Panc-Tu-I, Capan-1 and MIA PaCa-2 (refs 18, 19) resulted in a strongly reduced proliferation after 70–110 h (Fig. 3a), whereas treatment of the parental cell lines with doxycycline did not alter their proliferation (Supplementary Fig. 3).

This correlated with the growth inhibitory effects of both small-molecule inhibitors in these cell lines (Fig. 3b,c). Deltazinone 1 inhibited cell growth in a dose-dependent manner already observable at sub-μM concentrations. At doses higher than 3 μM decreasing cell indices indicated cell death ∼30 h after Deltazinone 1 administration in Panc-Tu-I cells and after ∼40 h in MIA PaCa-2 (Fig. 3b). In the Capan-1 cell line, Deltazinone 1 doses up to 24 μM led to strong growth inhibition but not cell death. This differential behaviour of the three KRas-dependent cell lines to PDEδ inhibition was reflected in the growth of these cells after doxycycline-induced PDEδ knockdown (Fig. 3a). In contrast to treatment with Deltazinone 1, a steep dose-dependent growth inhibitory effect and cell death was observed for Deltarasin in a concentration range from 3 to 5 μM in Panc-Tu-I, Capan-1 and MIA PaCa-2 cell lines (Fig. 3c,d)^13^. At 5 μM Deltarasin, detectable cell death in Panc-Tu-I occurred ∼9 h post treatment. The onset of Panc-Tu-I cell death after Deltazinone 1 treatment happened much later (∼13 h for 24 μM Deltazinone 1), while the onset was dependent on the dose of the drug (Fig. 3e). This indicates that Deltazinone 1 is less potent than Deltarasin in inhibiting the PDEδ/Arl2 system that restores KRas plasma membrane localization (see Discussion).

PANC-1 cells that harbour oncogenic KRas but are independent of this oncogene^18^, as well as the KRas wild-type-expressing BxPC-3 cancer cell line exhibited minor growth inhibitory effects on doxycycline-induced shRNA knockdown of PDEδ. Similarly, Deltazinone 1 doses up to 24 μM had little or no growth inhibitory effect on these cell lines (Fig. 3a,b). In contrast, Deltarasin concentrations above 9 μM resulted in an abrupt change in the RTCA profiles indicating rapid cell death of the KRas-independent cancer cell lines (Fig. 3c). These results point at unspecific cytotoxicity of Deltarasin at concentrations above 9 μM, which was not observed for Deltazinone 1 for concentrations up to 24 μM. The RTCA measurements were corroborated by long-term time-lapse microscopy that showed cell death for oncogenic KRas-dependent Panc-Tu-I cells treated with 5 μM Deltarasin or 10 μM Deltazinone 1, but not for oncogenic KRas-independent PANC-1 cells (Supplementary Movies 1–6).

### PDEδ inhibition decreases Ras-mediated signalling

**Figure 4:**
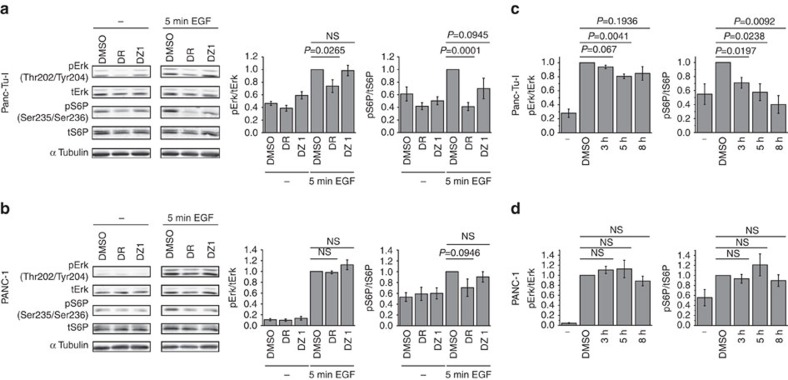
Effects of Deltarasin and Deltazinone 1 on Erk and S6P phosphorylation in human pancreatic ductal adenocarcinoma cell lines. Western blot analysis of Erk and S6P phosphorylation in KRas-dependent Panc-Tu-I (**a**,**c**) and KRas-independent PANC-1 cells (**b**,**d**). (**a**,**b**) Serum-starved cells were treated with vehicle control (DMSO), 5 μM Deltarasin (DR) or 20 μM Deltazinone 1 (DZ1) for 1 h. Phosphorylation levels were compared between unstimulated (−) and stimulated cells with 200 ng ml^−1^ EGF for 5 min (5 min EGF). Left panels: representative western blots of six independent experiments for each cell line. From top to bottom row: phosphorylated Erk on Thr202 and Tyr204 (pErk), total level of Erk (tErk), phosphorylated S6P on Ser235 and Ser236 (pS6P), total level of S6P (tS6P) and loading control (α-Tubulin). Right bar graphs: quantification of pErk/tErk±s.e.m. (left) and pS6P/tS6P±s.e.m. (right) normalized to EGF-stimulated DMSO control (DMSO). (**c**,**d**) Long-term treatment (3, 5 and 8 h) of cells with 20 μM Deltazinone 1, after which cells were stimulated with 200 ng ml^−1^ EGF for 5 min. Quantification of pErk/tErk±s.e.m. (left) and pS6P/tS6P±s.e.m. (right) normalized to EGF-stimulated DMSO control (DMSO). (−) Represent phosphorylation levels in unstimulated, vehicle control-treated cells. Values were obtained by averaging three (Panc-Tu-I) and four (PANC-1) independent experiments. *P* values were determined by applying Student's unpaired *t*-test. *P* values >0.4 are labelled as not significant (NS).

MAPK and mTOR pathways, which regulate proliferation and growth, are regulated by activity of proteins from the Ras family (Ras and Rheb, respectively)^15^,^20^. To examine whether PDEδ inhibition by Deltarasin and Deltazinone 1 downregulates the signal transduction via these pathways, we examined the phosphorylation of extracellular signal regulated kinase (Erk) and S6 Ribosomal Protein (S6P) in the oncogenic KRas-dependent Panc-Tu-1 and KRas-independent PANC-1 cells. Following treatment with Deltarasin or Deltazinone 1 for 1  h, phosphorylation levels of Erk and S6P were determined before and after 5 min EGF stimulation by quantitative western blot analysis (Fig. 4). As previously reported^13^, 5 μM Deltarasin reduced EGF-induced Erk phosphorylation in the KRas-dependent Panc-Tu-I cells, but not in PANC-1 cells (Fig. 4a,b), while the EGF-induced S6P phosphorylation was reduced in both lines (Fig. 4a,b). In contrast, 20 μM Deltazinone 1 administered for 1 h did not significantly affect Erk response to EGF in both cell lines, but reduced S6P phosphorylation in the KRas-dependent Panc-Tu-I cells (Fig. 4c). However, on longer incubation times (>3 h) with Deltazinone 1, EGF-induced Erk phosphorylation was reduced in Panc-Tu-I and S6P phosphorylation further decreased in a gradual manner, while PANC-1 cells, remained unaffected (Fig. 4c,d).

## Discussion

Deltarasin was the first small molecule effectively targeting the spatial organization of Ras by competing for the farnesyl-binding pocket of PDEδ and thereby resulting in a reduced proliferation of oncogenic KRas-dependent pancreatic ductal adenocarcinoma cells^13^. Herein, we compared the newly developed PDEδ inhibitor Deltazinone 1 based on the pyrazolopyridazinone scaffold to the benzimidazole-based Deltarasin and the genetic knockdown of PDEδ. Based on a crystal structure of pyrazolopyridazinone **2a** and molecular modelling, Deltazinone 1 has a similar PDEδ-binding mode as previously described for Deltarasin^13^.

The oncogenic KRas-dependent cell lines, Panc-Tu-I, Capan-1 and MIA PaCa-2 (refs 18, 19) exhibited reduced proliferation and even cell death after PDEδ knockdown and inhibitor treatments. We observed a high correlation between the effects of shRNA-mediated down-modulation and small-molecule inhibition of PDEδ on the proliferation pattern of the pancreatic cancer cell lines. However, Deltarasin exhibited cytotoxic effects at concentrations above 9 μM in all tested cell lines, causing a ‘switch-like' proliferation to cell death response. This is likely due to unspecific binding of Deltarasin to other proteins (Supplementary Fig. 2 and Supplementary Table 1), since this general cytotoxicity was not observed after Deltazinone 1 administration or PDEδ knockdown by shRNA. In contrast, Deltazinone 1 showed a more graded, dose-dependent inhibitory response on proliferation in oncogenic KRas-dependent cell lines and no general cytotoxicity could be observed at the concentrations tested.

Both Deltarasin and Deltazinone 1 exhibited an ‘in-cell' measured *K*_D_ of ∼60 nM for PDEδ, whereas μM concentrations of the inhibitors were required to affect the proliferation of KRas-dependent pancreatic cancer cells. Part of the explanation is the strong electrostatic interaction of KRas with negatively charged membranes that results in a very slow entropic leakage from the PM^7^. This means that PDEδ needs to be completely inhibited to relocalize KRas to endomembranes, as even a small pool of uninhibited, free PDEδ is sufficient to reinstate PM localization of KRas^5^.

The perinuclear localization of the Ras family protein Rheb depends solely on farnesylation as it lacks a second feature that will stabilize the interaction with membranes. For Rheb, the activity of the PDEδ-Arl2 ‘pump' is essential for maintenance of its localization, as it counters the tendency of this protein to distribute on all endomembranes within the cell. Due to the higher off-rate of Rheb from membranes as compared with KRas, even incomplete PDEδ inhibition will almost completely relocalize it to the extensive endomembrane surface across the whole cell^5^, leading to attenuation of mTOR activity, which is involved in regulation of cellular growth^21^. It is thus likely that at lower doses of the inhibitors, Rheb activity is impaired, whereas at saturating doses the activity of oncogenic KRas is affected.

This was reflected in Erk and S6P phosphorylation levels, as readouts of the Ras-regulated MAPK pathway and the Rheb-regulated mTOR pathway, respectively. The effects of both inhibitors were prominent in suppressing S6P phosphorylation in KRas-dependent cell lines, even after short incubation times (1 h). However, significant effects on Erk phosphorylation could only be observed after longer incubation time of Deltazinone 1. Deltarasin was more efficient in downregulating Erk phosphorylation in the KRas-dependent cell line, Panc-Tu-I, indicating a tighter interaction with PDEδ as compared with Deltazinone 1. We, however, measured similar affinities of both compounds for PDEδ (∼60 nM), which points to a more efficient displacement of Deltazinone 1 from PDEδ by Arl2 activity. This could lead to more available free PDEδ at steady state that can reinstate KRas PM localization. This could explain why higher doses of Deltazinone 1 than Deltarasin are needed to affect oncogenic KRas signalling to counter the allosteric displacement activity of Arl2.

The herein presented new PDEδ inhibitor chemotype caused specific cell death in KRas-dependent pancreatic cancer cell lines without exhibiting general cytotoxic effects for concentrations up to 24 μM. Deltazinone 1 represents a more specific second chemotype of PDEδ inhibitors and thereby resembles a non-cytotoxic compound for future medicinal chemistry studies. However, we note that pharmacological characterization of Deltazinone 1 revealed that this compound is rapidly metabolized in mice (see the Supplementary Fig. 4 and Supplementary Tables 2 and 3) making it non-suitable for *in vivo* experiments. Our results show that a combination of biochemical screening and structure-guided design enables the development of a novel inhibitor class for PDEδ with comparable potency but higher selectivity than the previously developed Deltarasin.

## Methods

### AlphaScreen

Screening based on Alpha technology was conducted in white, non-binding 1,536-well plates (Corning) in a final volume of 6 μl (ref. 13). For the screen, a mixture of His_6_-PDEδ, and biotinylated KRas-peptide (final concentrations 100 nM and 250 nM in HEPES 20 mM, 100 mM NaCl, 0.005% Chaps, pH 7.5) were added to the 1,536-well plates using a Multidrop Combi (Thermo Fisher Scientific) device. Compound solutions were directly added from 10 mM dimethylsulphoxide (DMSO) stock solutions to a final concentration of 10 μM using the acoustic dispenser Echo520 (Labcyte Inc.) and the resulting mixture was incubated for 30 min (for dose–response curves, compounds were tested at concentrations between 5 nM and 10 μM). Premixed Nickel Chelate Acceptor Beads and Streptavidin Donor Beads were added to a final concentration of 10 μg ml^−1^ using a Multidrop Combi (Thermo Fisher Scientific) device. The resulting mixture was incubated at 4 °C overnight. Plates were read on a Paradigm reader (Molecular Devices, Alphascreen 1536 HTS detection cartridge, temperature 29–33 °C).

### Displacement titrations for the determination of *K*_D_ values

Binding to PDEδ was validated and quantified by means of a displacement assay employing a fluorescent-tagged analogue of the HMG-CoA reductase inhibitor Atorvastatin (Lipitor), which has previously been shown to also bind to PDEδ^22^. The *K*_d_ values were determined by the fluorescence polarization competition-binding assay, which we developed before.

### Crystallography

Inhibitor **2a** was co-crystallized with PDEδ by mixing a solution of 500 μM small molecule with an equimolar solution of PDEδ resulting in 1% DMSO as a final concentration. Crystals were obtained from a Qiagen PEGs suite (0.2 M calcium acetate, 20% (w/v) PEG 3350). For flash freezing, the crystals in liquid nitrogen, we used cryoprotectant solution containing the mother liquor components in addition to glycerol. X-rays data were collected at the X10SA beamline of the Suisse Light Source, Villigen. XDS programme was used for processing the data. For solving the structures, molecular replacement was applied using the programme MolRep from the CCP4 software and PDEδ without bound farnesyl group, from the complex PDEδ-farnesylated Rheb complex (PDB 3T5G) as a search model. Further refinement of the model was performed using a combination of manual refinement (COOT programme) and the maximum likelihood restrained refinement (REFMAC5 programme). Final validation of the model using Ramachandran plot statistics showed none of the residues to be outliers (Supplementary Table 4).

### Molecular modelling

Molecular modelling experiments were carried out with the Maestro 9.1 suite (Schrödinger). Both ligand and receptor flexibility were taken into account by using receptor docking (Glide) in combination with the protein structure prediction embedded in the programme Prime. Protein Preparation Wizard (Schrödinger Maestro suite) was used to prepare the PDEδ co-crystal for the calculations. Previously obtained X-ray structures were used to define the active site. Water molecules were removed from the protein crystal structure, hydrogen atoms were added and resulting structure was refined by OPLS2005 force field. The minimization was ended when the RMSD reached 0.18 Å. Receptor Grid Preparation (Glide) was used to generate the protein grid that was subsequently utilized in docking experiments. The van der Waals radius scaling factor was set to 0.5 with a partial charge cutoff of 0.25. Arg61 was selected as a possible site for hydrogen bonding. In addition, docking runs were carried out using an additional hydrogen bond constraint with Tyr149. Ligand preparation for docking was carried out with LigPrep in Maestro 9.1 and the OPLS_2005 force field. Epika was used to generate possible states at target pH 7.0±4.0. Ligand-docking options in Glide were used for the first round of docking experiments. Under Setting, XP (extra precision), Dock flexibly, sample nitrogen inversions, sample ring conformation and Epik state penalties to docking score were selected, amide bonds were penalized for nonplanar conformation. Under the Ligands section, the Van der Waals radius scaling factor was set to 0.5 and the docking was set to match at least one out of two constraints. Several high-score binding poses were generated and co-crystallized compounds were re-docked and analysed in terms of overlay with the X-ray structure.

### Cell lines and culture conditions

The identity of all cancer cell lines (a kind gift from Stephan Hahn, Bochum) were tested by STR-DNA typing. Regular tests for the presence of mycoplasma were negative. Cells were cultured in DMEM supplemented with 10% fetal calf serum, 1% non-essential amino acids (PAN-Biotech), 1% L-glutamine (PAN-Biotech). Cells were maintained in a humidified incubator with 5% CO_2_ at 37 °C. For viral transduction, the packaging cell line HEK293T was seeded in 6 cm dishes and transfected next day with the vector pLKO-PDE6D-572 (ref. 13) using calcium phosphate precipitation. The following day, the supernatant was collected and used for viral transduction of the human pancreatic ductal adenocarcinoma cell lines. Selection with puromycine was done for all cell lines at a concentration between 0.4 and 1.0 μg ml^−1^ starting 1 day after transduction. Induction of the shRNA *PDE6D*-572 (ref. 13) expression was done with 200 ng ml^−1^ doxycycline. For live-cell microscopy, cells were cultured in four-well Lab-Tek chambers slides (Thermo Scientific/NUNC). Transfections were carried out with Effectene (Qiagen) or Lipofectamine 2000 (Invitrogen).

### Fluorescence lifetime imaging microscopy

Fluorescence lifetime images were acquired using a confocal laser-scanning microscope (FV1000, Olympus) equipped with a time-correlated single-photon counting module (LSM Upgrade Kit, Picoquant). For detection of the donor (mCitrine), the sample was excited using a 470-nm diode laser (LDH 470, Picoquant) at a 36-MHz repetition frequency. Fluorescence signal was collected through an oil immersion objective (× 60/1.35 UPlanSApo, Olympus) and spectrally filtered using a narrow-band emission filter (HQ 525/15, Chroma). Photons were detected using a single-photon counting avalanche photodiode (PDM Series, MPD) and timed using a single-photon counting module (PicoHarp 300, Picoquant).

### FLIM data analysis

Intensity thresholds were applied to segment the cells from the background fluorescence. Data were analysed via global analysis^23^ to obtain images of the molar fraction (*α*) of interacting mCitrine-Rheb with mCherry-PDEδ. Effects on *α* of Deltazinone 1 and Deltarasin was monitored by FLIM acquisition through a sequential addition of the compounds, followed by incubation for 10 min. Dose–response relationships were determined by plotting obtained *α* per addition, using equation:


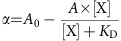


where *A*_0_ is *α* in the absence of the inhibitor, *A* an asymptotic offset and [X] concentration of the inhibitor in the medium. The *α* values before the inhibitor treatment were normalized to the value of 0.2.

### Real time cell analysis

RTCA was performed using 16-well E-plates on the Dual Plate xCELLigence instrument (Roche Applied Science, Indianapolis, IN). This system measures a dimensionless parameter called cell index, which evaluates the ionic environment at an electrode/solution interface and integrates information on cell number. Depending on the cell line 5 × 10^3^–1 × 10^4^ cells were plated in each well of the 16-well plates in 200 μl of cell culture medium. After seeding, cells were allowed to settle for 30 min at room temperature before being inserted into the xCELLigence instrument in a humidified incubator at 37 °C with 10% CO_2_. For RTCA experiments assaying cells stably expressing the inducible shRNA transgene, cells were seeded onto the E-plates in medium supplemented with 200 ng ml^−1^ doxycycline. For RTCA experiments, the cells were treated with Deltarasin or Deltazinone 1 after reaching a steady growth (24 h/48 h). The small-molecule inhibitors were added at indicated time points and in the indicated concentrations. The amount of DMSO was kept constant between the individual conditions and did not exceed 0.24 %. Continuous impedance measurements were then monitored every 15 min for up to 200 h. All assays were performed in duplicates. The cell index was normalized to 1 at the time point of drug administration. To display the inhibitor-dose growth-response relationship of Deltarasin and Deltazinone 1, the area below the curve was integrated for 60 h after administration of the drugs and normalized to the DMSO control.

### Western blots

Cells were lysed with RIPA-buffer (20 mM Tris pH 7.5, 150 mM NaCl, 0.5 mM Na_2_ EDTA, 1 % sodium deoxycholate, 0.1% sodium dodecyl sulfate, 1% IGEPAL supplemented with Complete Mini EDTA-free protease inhibitor (Roche Applied Science) and 1% phosphatase inhibitor cocktail 2 and 3 (P5726 and P0044, Sigma Aldrich), scraped off after 5 min on ice and centrifuged at 14,000*g* for 20 min at 4 °C. SDS–polyacrylamide gel electrophoresis was carried out with 10–25 μg of whole-cell lysate from each sample. The gels were blotted onto polyvinylidene difluoride membrane (Millipore) and blocked for 1 h at room temperature. Antibodies used for western blotting were: anti-PDE6D (Santa Cruz: sc-50260, 1:200), anti-Cyclophilin-B (Abcam: Ab160459, 1:3,000), p44/42 MAPK (Erk1/2) (3A7) (Cell Signaling: 9107, 1:1,000), Phospho-p44/42 MAPK (Erk1/2) (Thr202/Tyr204) (D13.14.4E) XP (Cell Signaling: 4370, 1:1,000), S6 Ribosomal Protein (54D2) (Cell Signaling: 2317, 1:500), Phospho-S6 Ribosomal Protein (Ser235/236) (2F9) (Cell Signaling: 4856, 1:1,000), α Tubulin (Sigma-Aldrich, T6074, 1:2,500) and matching secondary infrared antibodies IRDye 680 donkey anti rabbit IgG, IRDye 800 donkey anti mouse IgG, IRDye 800 donkey anti goat IgG (LI-COR, 1:10,000). Blots were scanned on a LI-COR Odyssey imaging system.

Western blots were quantified using ImageJ. The background of the blots was subtracted by applying the rolling ball subtraction with a radius of 50 pixels. Afterwards the integrated intensity of the bands was measured. Uncropped blots are shown in Supplementary Fig. 5.

## Additional information

**Accession codes:** The structure of compound **2a** bound to PDEδ has been deposited with the Protein Data Bank with ID 5E80.

**How to cite this article**: Papke, B. *et al*. Identification of pyrazolopyridazinones as PDEδ inhibitors. *Nat. Commun.* 7:11360 doi: 10.1038/ncomms11360 (2016).

## Supplementary Material

Supplementary Information Supplementary Figures 1-5, Supplementary Tables 1-4, Supplementary Methods and Supplementary References

Supplementary Movie 1 72 hour life cell imaging of PANC-1 cells treated with DMSO immediately before the beginning of the movie. Each frame represents one hour

Supplementary Movie 2 72 hour life cell imaging of Panc-Tu-I cells treated with DMSO immediately before the beginning of the movie. Each frame represents one hour.

Supplementary Movie 3 72 hour life cell imaging of PANC-1 cells treated with 5 μM Deltarasin immediately before the beginning of the movie.

Supplementary Movie 4 72 hour life cell imaging of Panc-Tu-I cells treated with 5 μM Deltarasin immediately before the beginning of the movie.

Supplementary Movie 5 72 hour life cell imaging of PANC-1cells treated with 10 μM Deltazinone 1 immediately before the beginning of the movie.

Supplementary Movie 6 72 hour life cell imaging of Panc-Tu-I cells treated with 10 μM Deltazinone 1 immediately before the beginning of the movie.

## Figures and Tables

**Table 1:**
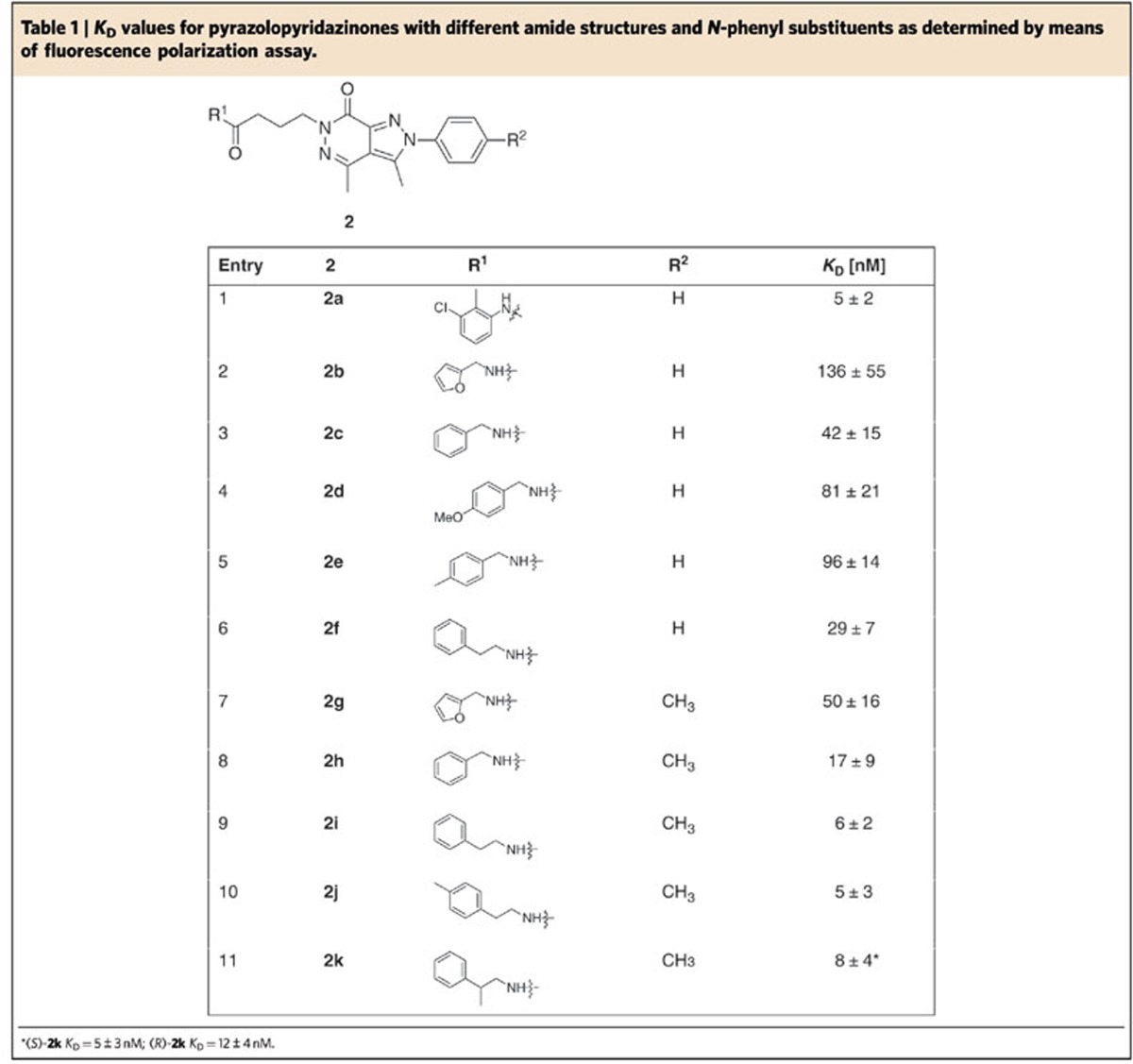
*K*_D_ values for pyrazolopyridazinones with different amide structures and *N*-phenyl substituents as determined by means of fluorescence polarization assay.

^*^(*S*)-**2k** *K*_D_=5±3 nM; (*R*)-**2k** *K*_D_=12±4 nM.

**Table 2:** Overview of pancreatic cancer cell lines used in this study including KRas mutation and oncogenic KRas-dependence.

**Cancer cell line**	**KRas mutation**	**Oncogenic KRas-dependence**
PANC-1	G12D	No^18^,^19^
BxPC3	wt	No^19^
Capan-1	G12V	Yes^18^,^19^
Panc-Tu-I/Pa-Tu-8902	G12V	Yes^18^
MIA PaCa-2	G12C	Yes^19^
