# Supplementary material for: Identification of pyrazolopyridazinones as PDEδ inhibitors
Source: Nat Commun. 2016 Apr 20;7:11360. doi: 10.1038/ncomms11360 (PMC4843002; doi:10.1038/ncomms11360)
Supplement: Supplementary Information — Supplementary Figures 1-5, Supplementary Tables 1-4, Supplementary Methods and Supplementary References [file ncomms11360-s7.pdf]

Supplementary Information

Supplementary Figures

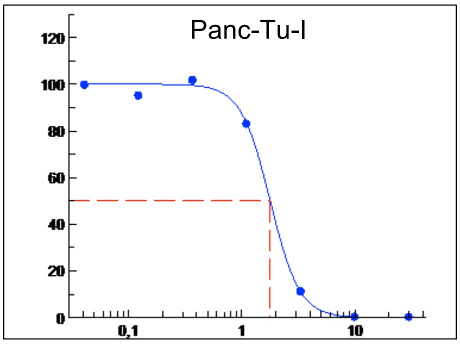

IC_{50} = 4.83 µM
(n = 14; Hill coefficient = -5.3)

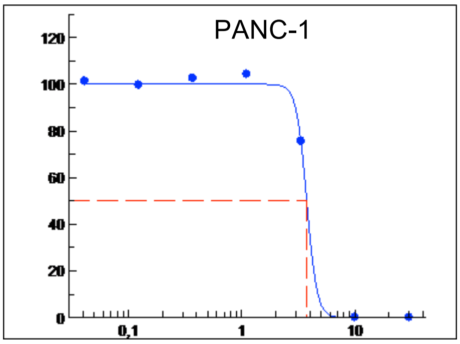

IC_{50} = 6.67 µM
(n = 10; Hill coefficient = -10.8)

Supplementary Figure 1: Antiproliferative activity of the KRas-PDEδ inhibitor Deltarasin (1a). Anti-proliferative activity of Deltarasin on human pancreatic ductal adenocarcinoma (hPDAC) cell lines harboring oncogenic KRas as determined by CellTiter-Glo® assay.

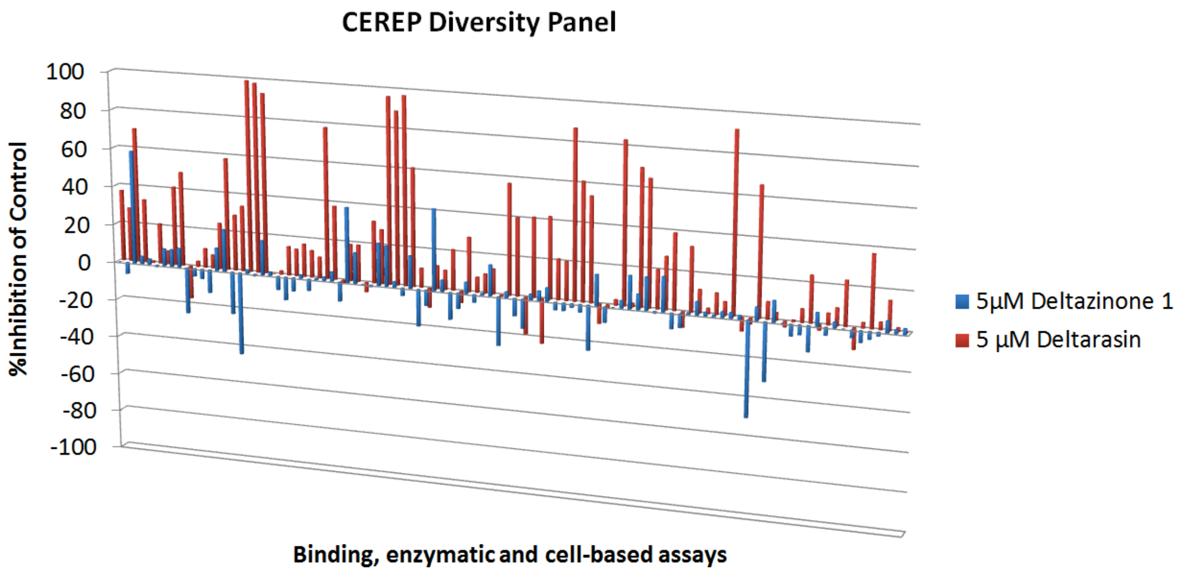

Supplementary Figure 2: Characterization of Deltarasin (1a) and Deltazinone 1 (2k) in a panel of biochemical and pharmacological assays. Deltarasin and Deltazinone 1 were tested at a concentration of 5 µM each in the CEREP diversity panel (Eurofins Pharma Discovery Services) including a comprehensive set of approximately 100 receptor binding, enzymatic and cell-based assays.

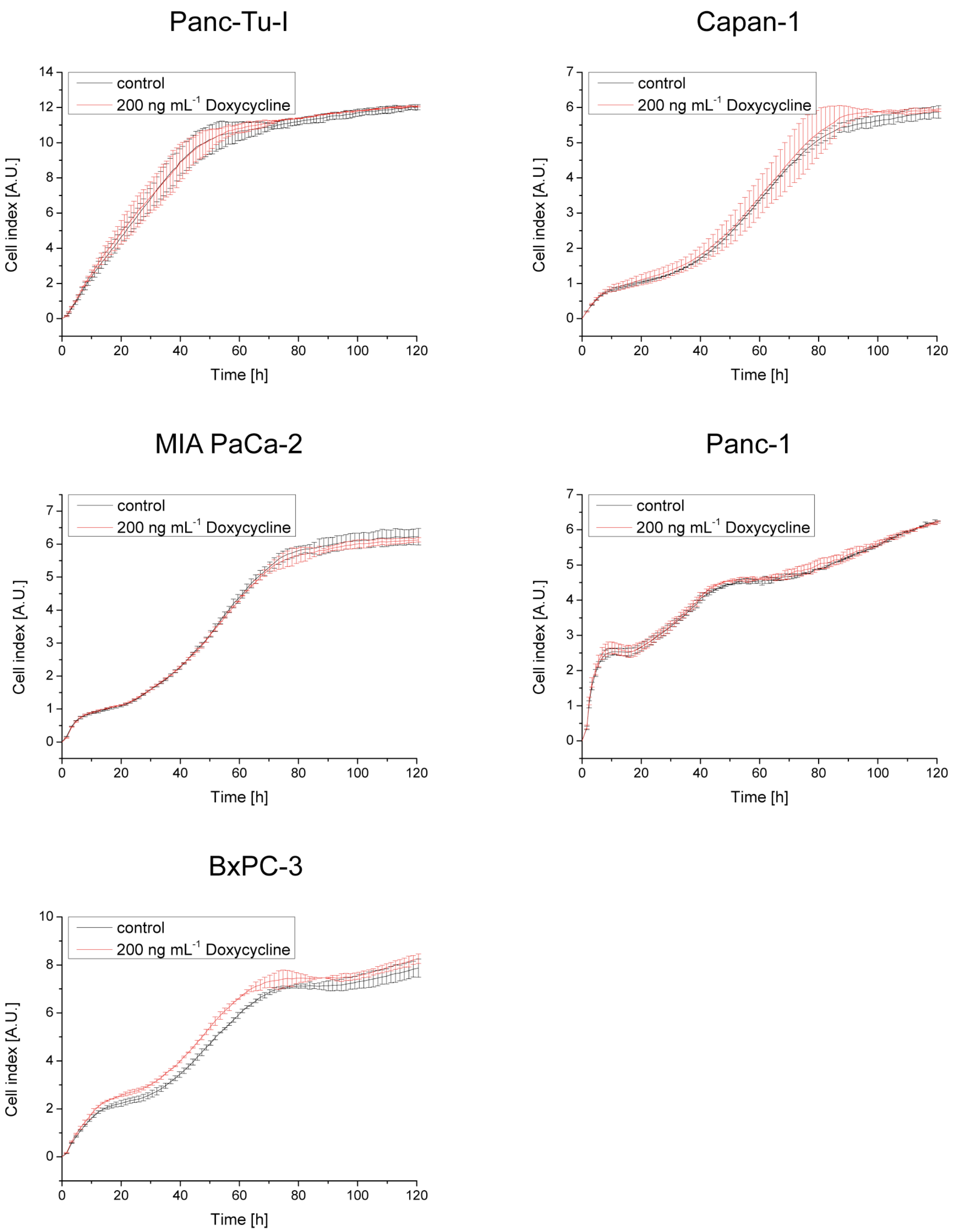

Supplementary Figure 3: Doxycycline does not influence cell growth of hPDAC cell lines. RTCA measurements of hPDAC cell lines in absence and presence of 200 ng mL^{-1} doxycycline, as used for shRNA expression.

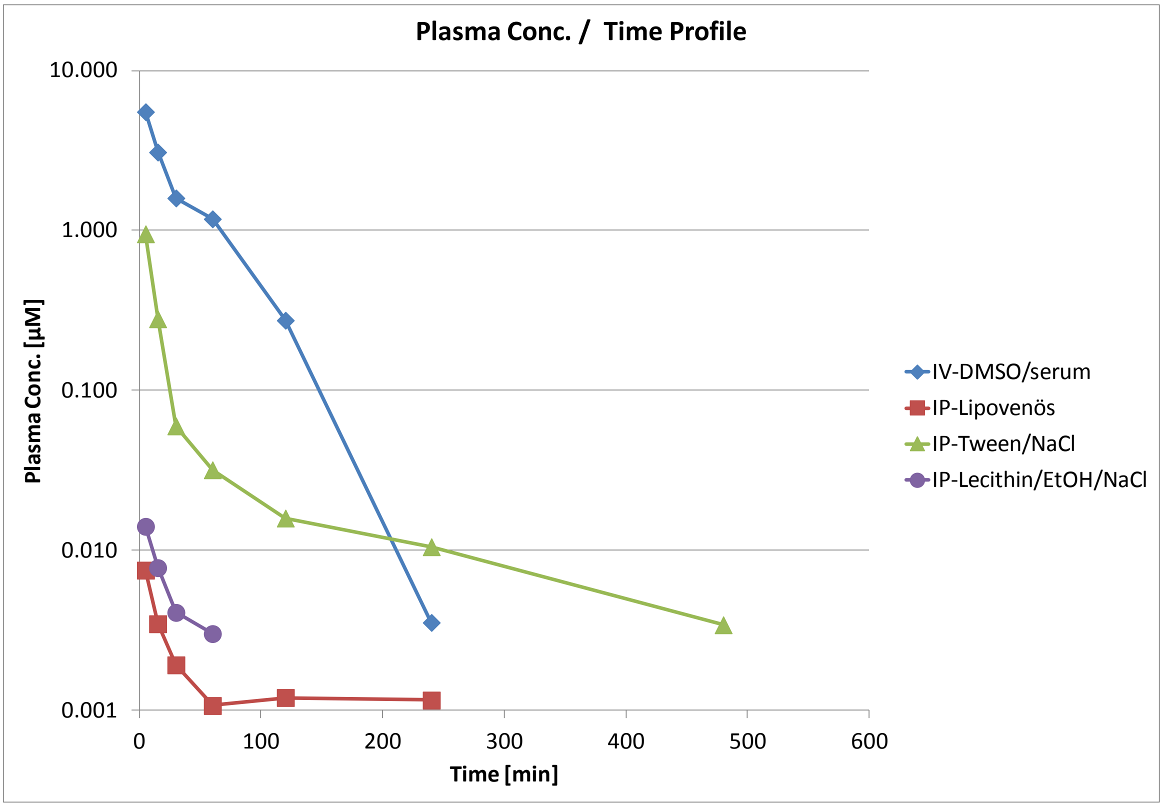

Supplementary Figure 4: Plasma concentration / time profile of Deltazinone 1 following intravenous or intraperitoneal administration in mice. NMRI mice were dosed with Deltazinone 1 at 3 mg kg^{-1} either intravenously (IV) or intraperitoneally (IP) following the formulation of the compound in 10% DMSO / 90% autologous serum (DMSO/serum), 100% Lipovenös, 10% PLR (Lipovenös) / 5% Tween80 / 50% physiological saline / 45% water (Tween/NaCl) or 5% lecithin / 5% ethanol / 50% physiological saline / 40% water (Lecithin/EtOH/NaCl). Blood samples were taken at different time points up to 24 h post-dose and analyzed for the total plasma concentration of Deltazinone 1 by LC-MS/MS analysis.

Insets Figure 3:

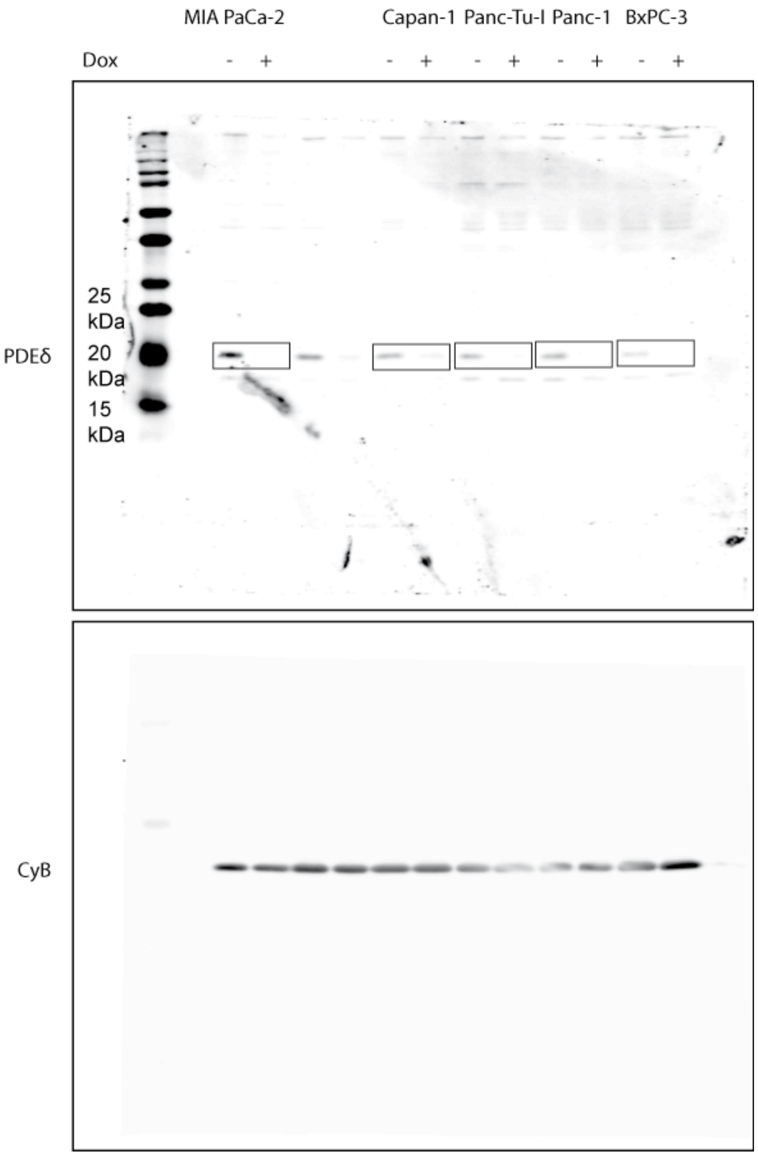

Insets 4a and b:

Panc- TU - I - western blot overview

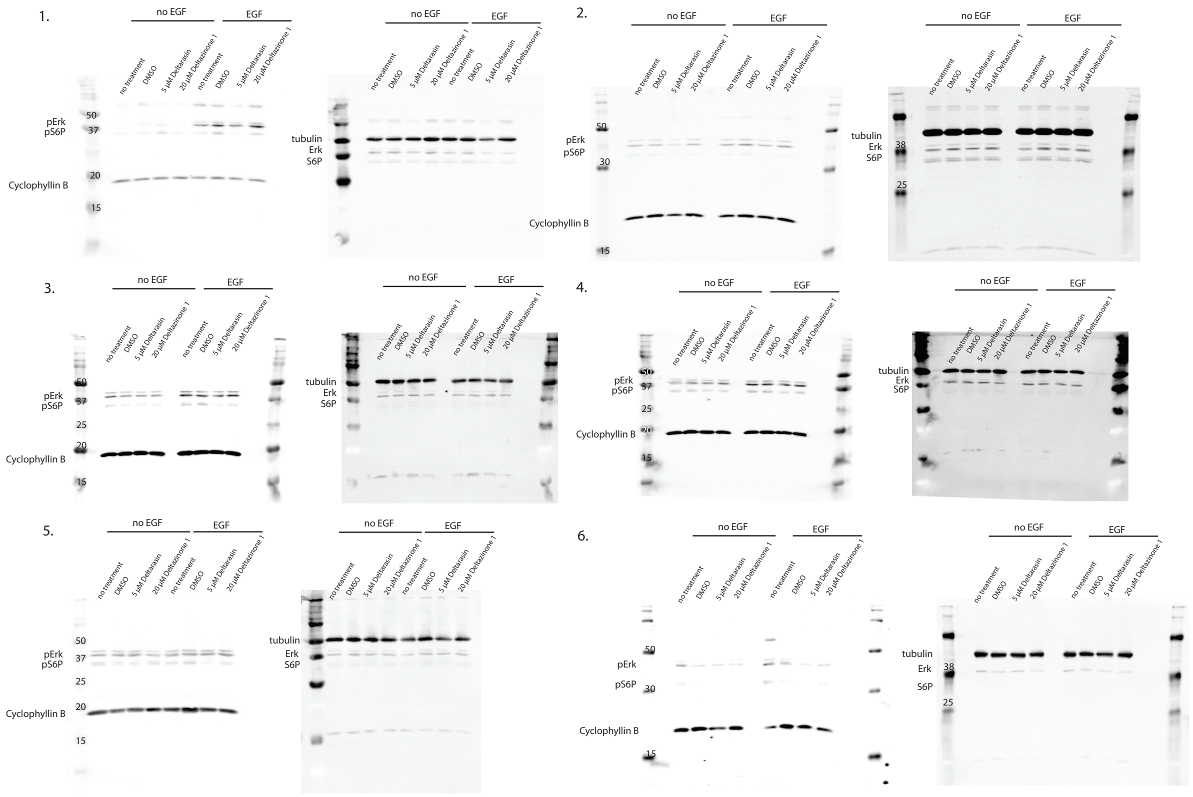

Panc 1- western blot overview

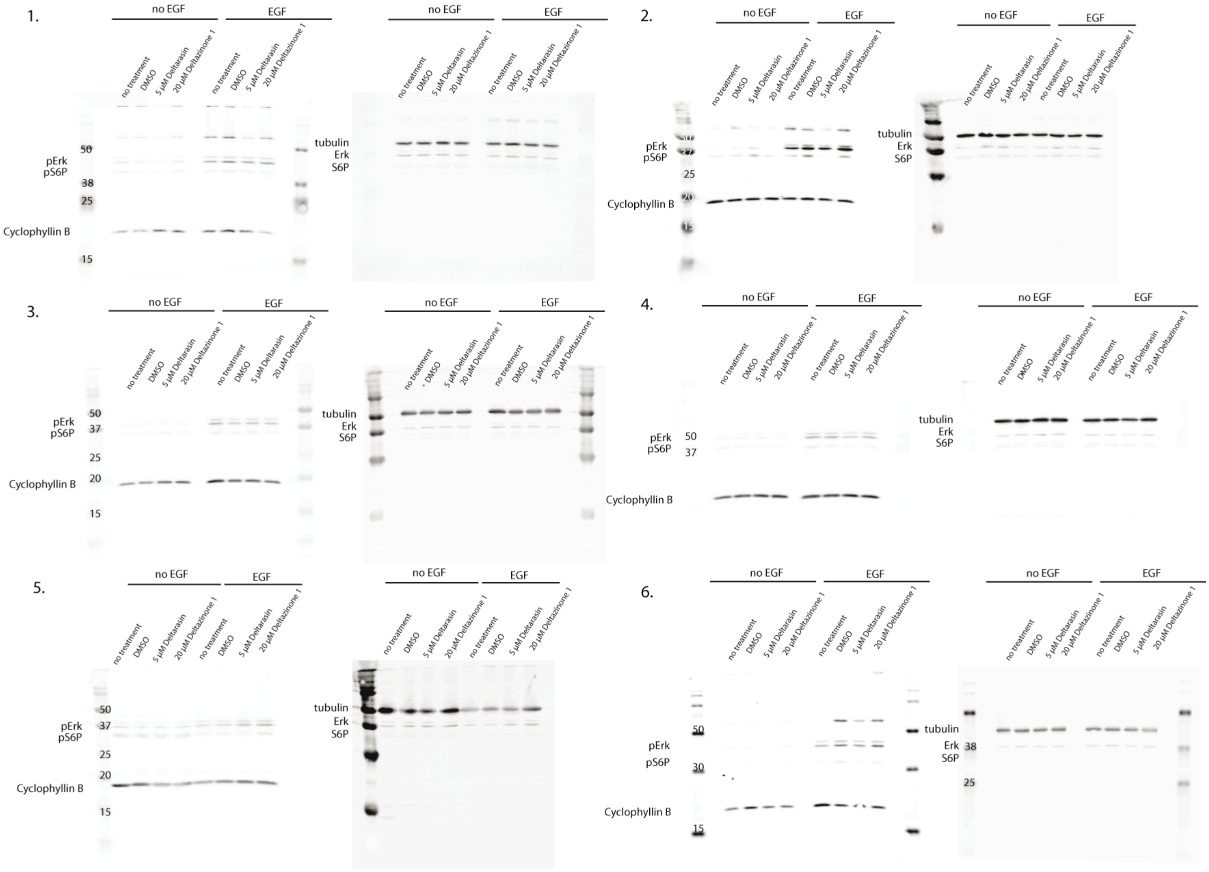

Insets Figure 4c and d:

Panc- TU - I - western blot overview

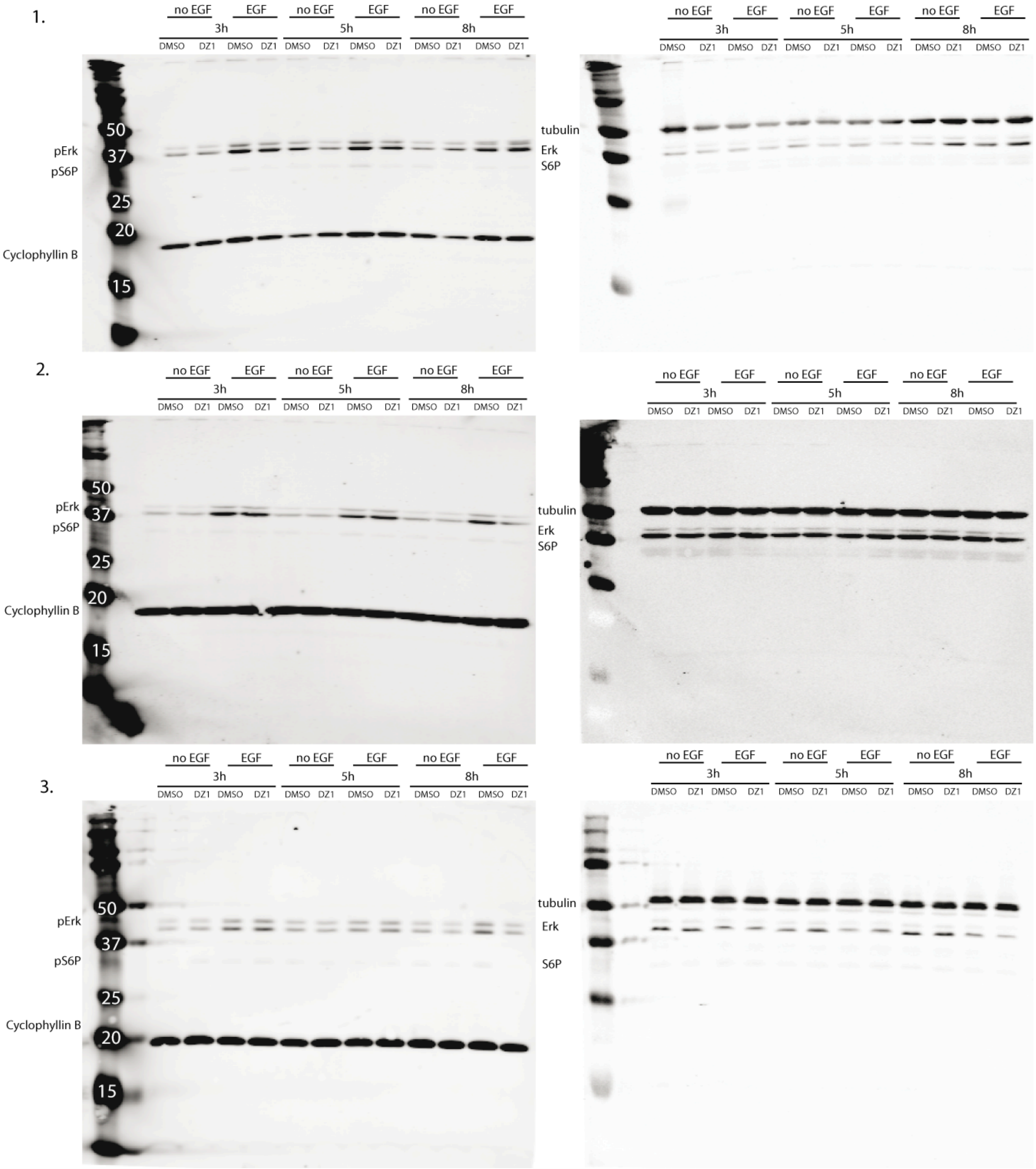

Panc 1 - western blot overview

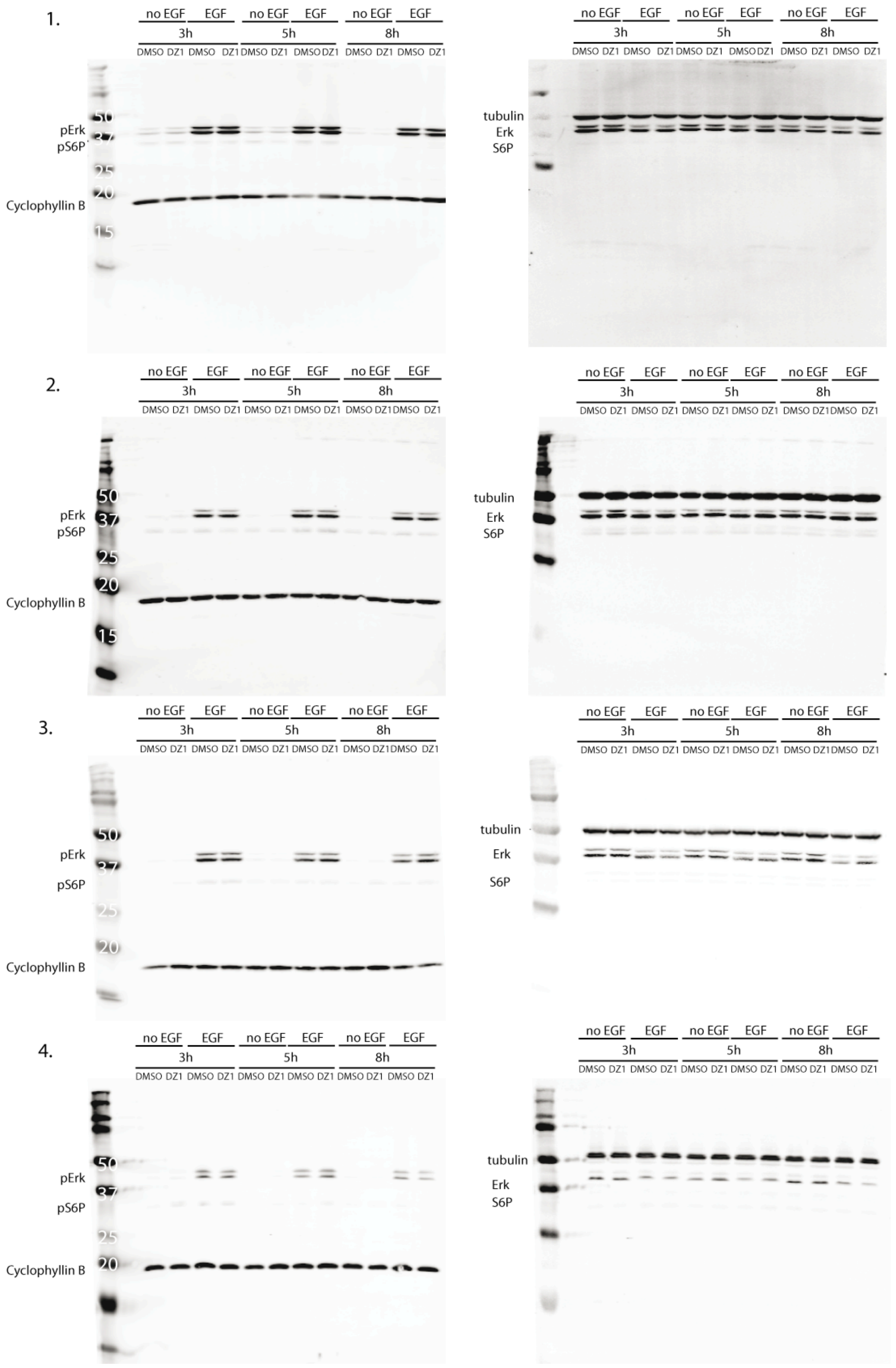

Supplementary Figure 5: Full Western blots used for insets and quantitative analysis in the main figures.

Supplementary Tables

Supplementary Table 1: Characterization of Deltarasin (**1a**) and Deltazinone 1 (**2k**) in a panel of biochemical and pharmacological assays (Cerep diversity panel, Eurofins Pharma Discovery Services).

**Test Concentration**	5 µM	5 µM
	**Deltarasin**	**Deltazinone 1**
**Binding Assays**	**% Inhibition of Control Specific Binding**	**% Inhibition of Control Specific Binding**
A1 (h) (antagonist radioligand)	37	0
A2A (h) (agonist radioligand)	28	-6
A3 (h) (agonist radioligand)	70	59
alpha 1 (non-selective) (antagonist radioligand)	33	4
alpha 2 (non-selective) (antagonist radioligand)	1	3
beta 1 (h) (agonist radioligand)	21	-1
beta 2 (h) (agonist radioligand)	7	9
AT1 (h) (antagonist radioligand)	41	9
AT2 (h) (agonist radioligand)	49	10
BZD (central) (agonist radioligand)	-17	-24
B1 (h) (agonist radioligand)	3	-4
B2 (h) (agonist radioligand)	10	-5
CB1 (h) (agonist radioligand)	7	-12
CB2 (h) (agonist radioligand)	24	12
CCK1 (CCKA) (h) (agonist radioligand)	58	22
CCK2 (CCKB) (h) (agonist radioligand)	29	-22
CRF1 (h) (agonist radioligand)	34	-43
D1 (h) (antagonist radioligand)	99	2
D2S (h) (antagonist radioligand)	98	-1
D3 (h) (antagonist radioligand)	93	18
D4.4 (h) (antagonist radioligand)	0	2
ETA (h) (agonist radioligand)	2	-7
ETB (h) (agonist radioligand)	15	-12
GABA (non-selective) (agonist radioligand)	14	-7
AMPA (agonist radioligand)	17	2
kainate (agonist radioligand)	14	-6
NMDA (antagonist radioligand)	11	1
H1 (h) (antagonist radioligand)	78	2
H2 (h) (antagonist radioligand)	38	5
H3 (h) (agonist radioligand)	-2	-10
I2 (antagonist radioligand)	19	39
BLT1 (LTB4) (h) (agonist radioligand)	19	16

CysLT1 (LTD4) (h) (agonist radioligand)	-5	0
MC4 (h) (agonist radioligand)	32	-1
MT1 (ML1A) (h) (agonist radioligand)	28	22
M (non-selective) (antagonist radioligand)	96	21
NK1 (h) (agonist radioligand)	89	3
NK2 (h) (agonist radioligand)	97	-4
NK3 (h) (antagonist radioligand)	61	17
Y (non-selective) (agonist radioligand)	10	-19
N neuronal alpha 4beta 2 (h) (agonist radioligand)	-10	-8
opioid (non-selective) (antagonist radioligand)	12	42
NOP (ORL1) (h) (agonist radioligand)	10	6
PPARgamma (h) (agonist radioligand)	21	-14
PCP (antagonist radioligand)	-6	-8
EP2 (h) (agonist radioligand)	28	6
IP (PGI2) (h) (agonist radioligand)	8	-4
P2X (agonist radioligand)	10	1
P2Y (agonist radioligand)	13	16
5-HT (non-selective) (agonist radioligand)	-1	-25
sigma (non-selective) (h) (agonist radioligand)	57	3
GR (h) (agonist radioligand)	40	-9
ER (non-selective) (h) (agonist radioligand)	-19	-15
PR (h) (agonist radioligand)	41	3
AR (h) (agonist radioligand)	-23	5
TRH1 (h) (agonist radioligand)	42	7
V1a (h) (agonist radioligand)	21	-4
V2 (h) (agonist radioligand)	20	-4
Ca2+ channel (L, dihydropyridine site) (antagonist radioligand)	87	-2
Ca2+ channel (L, diltiazem site) (benzothiazepines) (antagonist radioligand)	61	-4
Ca2+ channel (L, verapamil site) (phenylalkylamine) (antagonist radioligand)	54	-23
KATP channel (antagonist radioligand)	-10	16
KV channel (antagonist radioligand)	-2	-8
SKCa channel (antagonist radioligand)	3	0
Na+ channel (site 2) (antagonist radioligand)	83	4
Cl- channel (GABA-gated) (antagonist radioligand)	2	17
norepinephrine transporter (h) (antagonist radioligand)	70	8
dopamine transporter (h) (antagonist radioligand)	65	17
GABA transporter (antagonist radioligand)	20	-1
choline transporter (CHT1) (h) (antagonist radioligand)	27	18
5-HT transporter (h) (antagonist radioligand)	39	-8
**Enzyme and Cell-based Assays**	**% Inhibition of Control Values**	**% Inhibition of Control Values**
COX1 (h)	-8	-7
5-lipoxygenase (h)	33	1
PDE1B (h)	12	7

PDE2A1 (h)	3	2
PDE3A (h)	11	1
PDE4D2 (h)	7	3
PDE5 (h) (non-selective)	92	3
phosphatase 1B (h) (PTP1B)	-7	2
phosphatase CDC25A (h)	-3	-49
PKCalpha (h)	66	7
acetylcholinesterase (h)	9	-30
COMT (catechol- O-methyl transferase)	4	11
GABA transaminase	-3	0
MAO-A (h)	1	-6
MAO-B (h) recombinant enzyme	7	-5
tyrosine hydroxylase	24	-13
ATPase (Na+/K+)	-3	7
CENP-E (h)	6	-4
Eg5 (h)	9	3
HDAC3 (h)	23	0
HDAC4 (h)	-11	-4
HDAC6 (h)	3	-6
HDAC11 (h)	37	-4
sirtuin 1 (h) (inhibitor effect)	4	-2
sirtuin 2 (h) (inhibitor effect)	15	6
adenylyl cyclase (activator effect)	2	2
guanylyl cyclase (h) (activator effect)	0	3

Supplementary Table 2: Compilation of pharmacokinetic parameters following the administration of Deltazinone 1 to NMRI mice. (t1/2 = half life; Cmax = maximum concentration; Tmax = time to reach Cmax; C0 = extrapolated concentration after intravenous administration at time zero; AUC 0-t = area under the plasma concentration-time curve calculated from 0 to the last measured concentration; AUC 0-inf = AUC from 0 to infinity; Cl = total plasma clearance; Vss = steady-state volume of distribution; %F = bioavailability)

Parameter	Unit	Value	Value	Value	Value
Mouse strain		female NMRI	female NMRI	female NMRI	female NMRI
Route		IV	IP	IP	IP
Formulation		DMSO/ serum	Lipovenös	Tween/NaCl	Lecithin/ EtOH/NaCl
Dose	mg kg^{-1}	3	3	3	3
t1/2	h	0.35	2.02	2.67	0.43
Cmax	ng mL^{-1}	NA	3.44	435.69	6.46
Tmax	h	NA	0.08	0.08	0.08
C0	ng mL^{-1}	3375.67	NA	NA	NA
AUC 0-t	h ng mL^{-1}	1619.04	2.81	130.92	2.60
AUC 0-inf	h ng mL^{-1}	1619.86	4.35	136.96	3.46
Cl	L h^{-1} kg^{-1}	1.85	NA	NA	NA
Vss	L kg^{-1}	1.18	NA	NA	NA
%F	%	NA	0.30	8.50	0.20

Supplementary Table 3: Metabolic stability in murine liver microsomes of Deltazinone 1. (Cl_{int} = in vitro intrinsic clearance)

**Metabolic stability in murine liver microsomes**	**Deltazinone 1**	**Classification**
Cl_{int}	1760 µl min^{-1} mg^{-1}	Highly unstable

Supplementary Table 4: Data collection and refinement statistics of the crystal structure of compound 2a bound to PDEδ (PDB code: 5E80)

	**2a**
**Data collection**	
Space group	P 1
Cell dimensions	
a, *b*, *c* (Å)	31.76, 40.90, 68.80
A, β, γ (°)	97.70,102.38, 89.31
Resolution (Å)	2.60
R_{merge}	11.3 (46.6)
$I / \sigma I$	9.45 (3.26)
Completeness (%)	97.7 (96.7)
Redundancy	3.45 (3.40)
**Refinement**	
Resolution (Å)	2.60
No. reflections	9570
R_{work} / R_{free}	18.13 / 25.57
No. atoms	
Protein	2432
Ligand/ion	64

Water	16
B-factors	
Protein	37.7
Ligand/ion	40.2
Water	37.3
R.m.s. deviations	
Bond lengths (Å)	0.013
Bond angles (°)	1.732

Values in parentheses correspond to the high resolution shell

Supplementary Methods

Biochemical and Pharmacological Assays

Determination of cell viability using CellTiter-Glo®

Cells were seeded on day 1 at 500 cells per well to assure assay linearity and optimal signal intensity. After incubation for 24h in humidified incubator at 37°C, 5% CO_2, compounds / DMSO were added at different concentrations. Cells were further incubated for 72 h at 37°C and 5% CO_2. Cells treated with the compound vehicle (DMSO) were used as positive controls and cells treated with 10 µM Staurosporine served as negative controls.

At day 5 the CellTiter-Glo® Reagent was prepared according to the instructions of the manufacturer (Promega): The Reagent was mixed 1:1 with cell culture medium. Thereon, mixture and assay plates were equilibrated at room temperature for 20 min. Equal volumes of the reagent-medium-mixture were added to the volume of culture medium present in each well. The plates were mixed at 200 rpm for 2 minutes on an orbital shaker and afterwards incubated at room temperature for 10 minutes. Following incubation the luminescence was recorded on a Victor microplate reader (Perkin Elmer) using a 200 ms integration time. The data was analyzed with Excel using the XLFIT Plugin (dose response Fit 205) for IC_{50}-determination.

As quality control the Z´-factor was calculated from 16 positive and negative control values. Only assay results showing a Z´-factor ≥ 0.5 were used for further analysis.

CEREP-panel

To examine the selectivity of both PDEδ inhibitors, Deltarasin and Deltazinone 1 were tested side by side at a concentration of 5 µM each in the CEREP diversity panel (Eurofins Pharma Discovery Services) encompassing a comprehensive set of ca. 100 receptor binding, enzymatic and cell-based assays. With the exception of one assay (59% inhibition of specific control binding to adenosine A3 receptor), Deltazinone 1 did not show in any other assay an inhibition or stimulation of higher than 50% which is considered to represent a significant effect of the test compound (Supplementary Table 1). In contrast, Deltarasin showed high binding affinity to e.g. dopamine receptor isoforms, various ion channels and transporters suggesting that Deltarasin unspecifically binds to and eventually modulates several off-targets (Supplementary Table 1).

Pharmacokinetic profiling of Deltazinone 1 (2k)

Determination of pharmacokinetic properties in vivo

Deltazinone 1 was administered to female NMRI mice at an intravenous dose of 3 mg kg^{-1} in 10% DMSO / 90% autologous serum or at an intraperitoneal dose of 3 mg kg^{-1} in either 100% Lipovenös, 10% PLR (Fresenius Kabi, Bad Homburg, Germany) or 5% Tween80 / 50% physiological saline / 45% water or 5% lecithin / 5% ethanol / 50% physiological saline / 40% water. Blood samples were collected up to 24 h post-dose from the tail vein into tubes containing heparin sodium as an anticoagulant and centrifuged at 1500 g for 5 minutes at 4°C. Prior to LC-MS/MS analysis, plasma proteins were precipitated with acetonitrile containing an internal standard and samples were filtered. A calibration curve was obtained from spiked blank plasma samples. Deltazinone was measured using a Shimadzu UPLC system connected to a QTrap 4000 hybrid triple quadrupole/linear ion trap mass spectrometer (AB Sciex).

The regression equation of the calibration curve was used to calculate plasma concentrations. Pharmacokinetic parameters were calculated using the PKSolver software[2].

Animal experiments and care were conducted at Synovo GmbH (Tübingen, Germany) in accordance with the guidelines of institutional authorities and approved by local authorities.

Determination of metabolic stability in murine liver microsomes

Metabolic stability of Deltazinone 1 at a final concentration of 1 µM was determined by incubation with liver microsomes derived from mice. Compound depletion was measured over time of 50 minutes by LC-MS/MS in order to calculate compound half-life $t_{1/2}$. Conversion to the in vitro intrinsic clearance CL_{int} expressed in [µl min^{-1} mg^{-1}] was performed using the following equation: Cl_{int} [µl min^{-1} mg^{-1}] = (0.693/$t_{1/2}$[min]) x (reaction volume [µl]/microsomal protein [mg]).

Chemical Synthesis

General Information:

All reactions involving air- or moisture-sensitive reagents or intermediates were carried out in flame-dried glassware under an argon atmosphere. Dry solvents (THF, toluene, MeOH, DMF) were used as commercially available; CH_2Cl_2 was purified by the Solvent Purification System M-BRAUN Glovebox Technology SPS-800. Analytical thin-layer chromatography (TLC) was performed on Merck silica gel aluminium plates with F-254 indicator. Compounds were visualized by irradiation with UV light or potassium permanganate staining. Column chromatography was performed using silica gel Merck 60 (particle size 0.040-0.063 mm). ^{1}H-NMR and

^{13}C-NMR were recorded on a Bruker DRX400 (400 MHz), Bruker DRX500 (500 MHz) and INOVA500 (500 MHz) at 300 K using $CDCl_3$ or $(CD_3)_2SO$ as solvents. All resonances are reported relative to TMS. Spectra were calibrated relative to solvent's residual proton and carbon chemical shift: $CDCl_3$ (δ = 7.26 ppm for ^1H NMR and δ = 77.16 ppm for ^{13}C NMR); $(CD_3)_2SO$: δ= 2.50 ppm for ^1H NMR and δ= 39.52 ppm for ^{13}C NMR). Multiplicities are indicated as: br s (broadened singlet), s (singlet), d (doublet), t (triplet), q (quartet), quin (quintet), m (multiplet); and coupling constants (J) are given in Hertz (Hz). High resolution mass spectra were recorded on a LTQ Orbitrap mass spectrometer coupled to an Acceka HPLC-System (HPLC column: Hypersyl GOLD, 50 mm × 1 mm, particle size 1.9 μm, ionization method: electron spray ionization). Atorvastatin was purchased from Sequoia Reseach Products. Compounds **2a**-**2j** were purchased from chemdiv, but they can easily be prepared by following the synthetic sequence and procedure described below. All other chemicals and solvents were purchased from Sigma-Aldrich, Fluka, TCI, Acros Organics, ABCR and Alfa Aesar. Unless otherwise noted, all commercially available compounds were used as received without further purifications.

Synthesis of pyrazolopyridazinone acid precursor 7

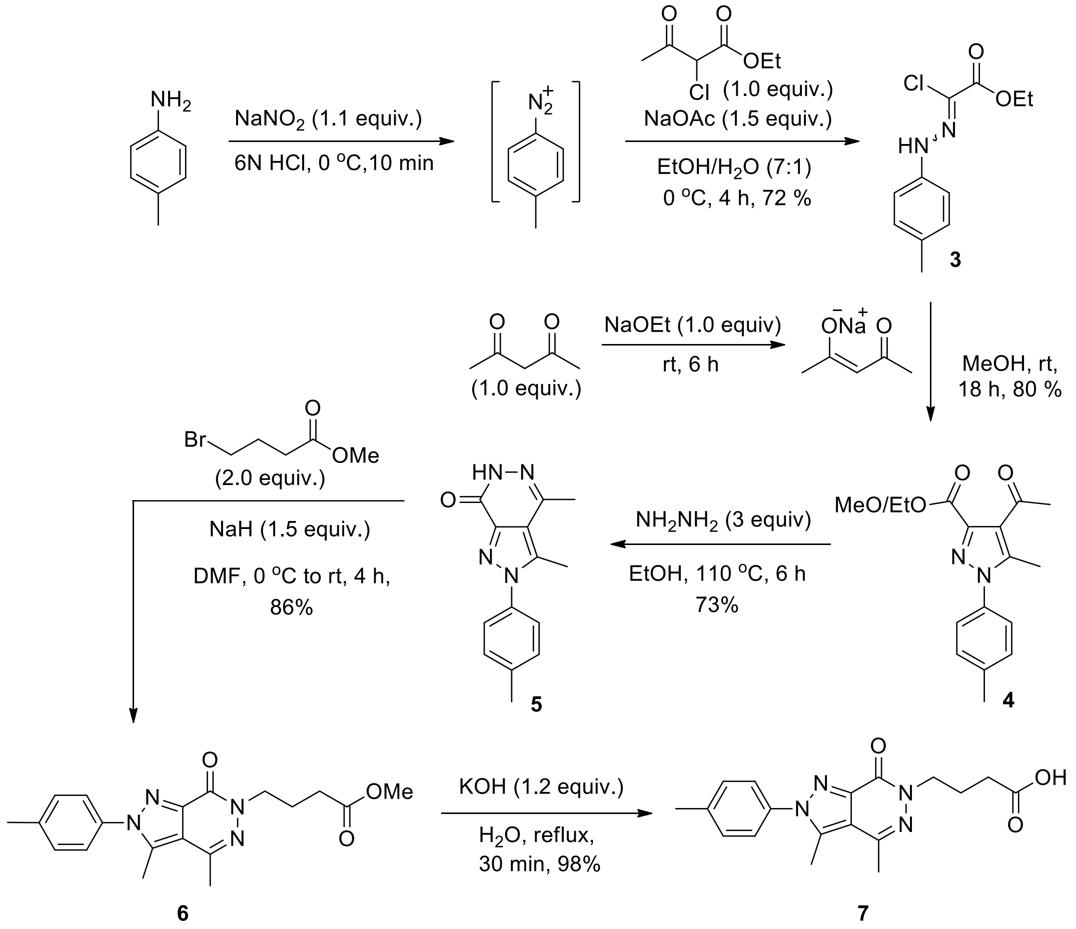

Ethyl 2-chloro-2-(2-(p-tolyl)hydrazono)acetate (**3**) [3]

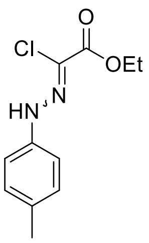

A solution of aniline derivative (20 mmol) in dilute HCl (1:1, 14 mL) was cooled to 0 °C, and a cold solution of $NaNO_2$ (1.1 equiv.) in H_2O (19 mL) was added drop wise over 15 min while maintaining internal solution temperature below 5 °C. After addition, the reaction mixture was stirred for another 10 min keeping the internal temperature below 0 °C. The resulting ice-cold solution of diazonium derivative was then added drop wise *via* cannula to a precooled (0 °C) solution of ethyl-2-chloroacetoacetate (1.0 equiv.) and NaOAc (1.5 equiv.) in H_2O/EtOH (1:7, 80 mL). After complete addition, the reaction mixture was stirred for 4 h at the same temperature and then was quenched by addition of 200 mL of cold water. The resultant precipitate was filtered and dried under vacuum to furnish the desired product (3.50 g, 72%) as an off-white solid. ^{1}H NMR (400 MHz, $CDCl_3$) δ 8.34 (s, 1H), 7.13 (s, 4H), 4.38 (q, *J* = 7.1 Hz, 2H), 2.31 (s, 3H), 1.40 (t, *J* = 7.1 Hz, 3H). ^{13}C NMR (101 MHz, $CDCl_3$) δ 159.9, 139.4, 132.8, 130.0, 115.4, 114.2, 62.8, 20.8, 14.4. HRMS: calc. for $[M+H]^+$ $C_{11}H_{14}O_2N_2Cl$: 241.07383, found: 241.07342.

3,4-Dimethyl-2-(p-tolyl)-2H-pyrazolo[3,4-d]pyridazin-7(6H)-one (**5**) [3]

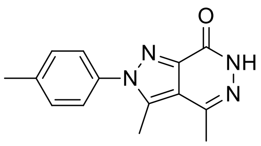

2,4-Pentanedione (1.34 mL, 13.0 mmol, 1 equiv) was added drop wise to a solution of NaOEt (21% wt in EtOH, 4.85 mL, 13 mmol, 1.0 equiv.) in anhydrous MeOH (10 mL) at ambient temperature and the reaction mixture was stirred for 4 h. Then the corresponding solid hydrazonyl chloride **3** (3.13 g, 13 mmol, 1.0 equiv) was added in portions and the reaction was left to stir for 16 h. After completion, the volatile components were removed and the crude material was re-dissolved in dichloromethane (30 mL). The organic layer was then sequentially washed with H_2O (10 ml × 2), saturated brine (15 mL), dried over Na_2SO_4, filtered and concentrated in *vacuo* to give crude **4** (**4** was isolated as a mixture of ethyl and methyl ester, which was confirmed by GC-MS. Transesterification was observed as MeOH was used as solvent. Reaction works equally well in EtOH. So trans-esterification can be avoided by using NaOEt as base in combination with anhydrous EtOH as solvent). Crude **4** was subjected to the next step without further purification.

4 (3.75 g, 13.0 mmol) and hydrazine monohydrate (1.91 mL, 39.3 mmol, 3.0 equiv.) were dissolved in EtOH (20 mL) and the mixture was heated in a sealed tube at 110 °C for 6 h. After completion, the reaction mixture was allowed to come to room temperature and then was cooled in an ice-bath. The resulting precipitate was filtered, washed with water and

dried under vacuum to yield the desired product **5** (2.4 g, 9.44 mmol, 72% over two step) as a grey solid. ^{1}H NMR (500 MHz, DMSO) δ 11.98 (s, 1H), 7.49 (d, *J* = 8.2 Hz, 2H), 7.43 (d, *J* = 8.2 Hz, 2H), 2.59 (s, 3H), 2.49 (s, 3H), 2.43 (s, 3H). ^{13}C NMR (126 MHz, DMSO) δ 156.4, 141.2, 141.0, 139.2, 137.1, 135.9, 129.8, 125.7, 117.4, 20.7, 19.2, 11.8. HRMS: calc. for $[M+H]^+$ $C_{14}H_{15}ON_4$: 255.12404, found: 255.12345.

Methyl 4-(3,4-dimethyl-7-oxo-2-(p-tolyl)-2H-pyrazolo[3,4-d]pyridazin-6(7H)-yl)butanoate (**6**)

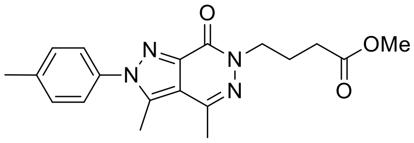

NaH (60 % in mineral oil, 0.236 g, 5.89 mmol, 1.50 equiv.) was added in small portions to a solution of **5** (1.0 g, 3.93 mmol, 1.0 equiv) in DMF (3 mL) at 0 °C and the reaction mixture was stirred for an hour at this temperature. Then a solution of methyl 4-bromobutanoate (0.99 mL, 7.86 mmol, 2.0 equiv) in DMF (0.5 mL) was added dropwise and the resulting reaction mixture was stirred for an additional 4 h at room temperature. After completion, the reaction was quenched by adding H_2O (20 mL) and diluted with EtOAc (20 mL). The aqueous layer was extracted with EtOAc (2 × 10 mL); combined organic layers were sequentially washed with H_2O (2 x 10 mL), brine (2 × 10 mL), dried over Na_2SO_4, filtered, and concentrated under vacuum. The crude residue was purified by flash column chromatography using 4% MeOH/CH_2Cl_2 as an eluent to give the desired product **6** (1.2 g, 3.39 mmol, 86%) as a white solid. ^{1}H NMR (400 MHz, $CDCl_3$) δ 7.34 (d, *J* = 8.4 Hz, 2H), 7.30 (d, *J* = 8.4 Hz, 2H), 4.22 (t, *J* = 6.9 Hz, 2H), 3.65 (s, 3H), 2.61 (s, 3H), 2.53 (s, 3H), 2.43 (s, 3H), 2.40 (t, *J* = 8.0 Hz , 2H), 2.15 (quin, *J* = 7.1 Hz, 2H). ^{13}C NMR (101 MHz, $CDCl_3$) δ 173.6, 156.2, 142.0, 140.9, 139.7, 136.3, 136.0, 129.9, 125.9, 125.8, 117.7, 51.6, 48.8, 31.3, 24.1, 21.3, 19.9, 12.3. HRMS: calc. for $[M+H]^+$ $C_{19}H_{23}O_3N_4$: 355.17647, found: 355.17677.

4-(3,4-dimethyl-7-oxo-2-(p-tolyl)-2H-pyrazolo[3,4-d]pyridazin-6(7H)-yl)butanoic acid (**7**)

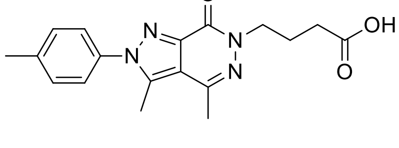

A heterogeneous mixture of pyrazolopyridazone derivative **6** (1.1 g, 3.10 mmol, 1.0 equiv) and KOH (0.21 g, 3.72 mmol, 1.2 equiv) in H_2O (15 mL) was heated to reflux until the entire solid had dissolved (30 min). Then the reaction mixture was allowed to come to room temperature, cooled to 0 °C and was acidified with 10% aqueous HCl solution to a pH of 5-6. The resultant precipitate was then filtered, dried

under vacuum to yield analytically pure **7** as a white solid (1.03 g, 3.03 mmol, 98%). ^{1}H NMR (400 MHz, DMSO) δ 12.09 (s, 1H), 7.47 (d, *J* = 8.2 Hz, 2H), 7.41 (d, *J* = 8.2 Hz, 2H), 4.06 (t, *J* = 6.9 Hz, 2H), 2.58 (s, 3H), 2.50 (s, 3H), 2.41 (s, 3H), 2.25 (t, *J* = 7.3 Hz, 2H), 1.92 (quin, *J* = 7.1 Hz, 2H). ^{13}C NMR (101 MHz, DMSO) δ 174.0, 155.2, 141.1, 141.1, 139.3, 137.4, 135.9, 129.9, 125.7, 117.1, 48.2, 30.8, 23.8, 20.8, 19.4, 11.9. HRMS: calc. for $[M+H]^+$ $C_{18}H_{21}O_3N_4$: 341.16082, found: 341.16096.

General procedure I (GP-I) for amide coupling

To a solution of acid derivative **7** (0.049 g, 0.15 mmol, 1.0 equiv) and DMAP (0.024 g, 0.195 mmol, 1.3 equiv) in THF/DCM (1:1, 0.6 mL and 0.6 mL) was added EDC·HCl (0.037 g, 0.195 mmol, 1.3 equiv), followed by the corresponding amine derivative (0.158 mmol, 1.05 equiv) and the resulting solution was stirred for 16 h at ambient temperature. Once completed, as observed by TLC, the reaction mixture was diluted with CH_2Cl_2 (5 mL) and quenched by adding saturated $NaHCO_3$ solution (5 mL). The aqueous layer was extracted with dichloromethane (2 × 5 mL) and combined organic layers were sequentially washed with saturated NH_4Cl solution (5 mL × 2), H_2O (5 ml × 2) and brine (5 mL); dried over Na_2SO_4, filtered and concentrated under vaccum. The crude residue was further purified by FCC on silica-gel using 3.5% MeOH/CH_2Cl_2 as an eluent to afford desired amide compounds **2**.

4-(3,4-dimethyl-7-oxo-2-(p-tolyl)-2H-pyrazolo[3,4-d]pyridazin-6(7H)-yl)-N-(2-phenylpropyl)butanamide (**2k**)

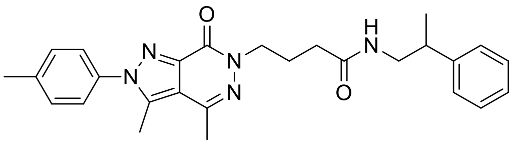

The title compound (1.20 g, 2.62 mmol, 94%) was prepared according to GP-I by treating acid derivative **7** (0.95 g, 2.79 mmol) with β-methylphenethylamine (0.426 mL, 2.93 mmol), EDC·HCl (0.696 g, 3.63 mmol) and DMAP (0.44 g, 3.628 mmol) in a DCM/THF (9/9 mL) solvent mixture. ^{1}H NMR (400 MHz, $CDCl_3$) δ 7.40 – 7.31 (m, 4H), 7.30 – 7.24 (m, 4H), 7.23 – 7.16 (m, 1H), 4.11 – 3.97 (m, 2H), 3.58 – 3.49 (m, 1H), 3.48 – 3.39 (m, 1H), 3.09 (sex, *J* = 7.1 Hz, 1H), 2.64 (s, 3H), 2.54 (s, 3H), 2.46 (s, 3H), 2.17 (t, *J* = 6.5 Hz, 2H), 2.12 – 2.02 (m, 2H), 1.31 (d, *J* = 7.0 Hz, 3H). ^{13}C NMR (101 MHz, $CDCl_3$) δ 173.2, 157.3, 144.9, 142.2, 142.1, 140.3, 136.8, 136.3, 130.3, 128.9, 127.7, 126.8, 126.2, 118.1, 48.8, 46.7, 40.0, 33.6, 26.1, 21.7, 20.3, 20.1, 12.7. HRMS: calc. for $[M+H]^+$ $C_{27}H_{32}O_2N_5$: 458.25505, found: 458.25514.

The enantiomers of Deltazinone 1 (**2k**); (*R*)-Deltazinone (**2k-*R***) and (*S*)-Deltazinone (**2k-*S***) were synthesized by reacting acid derivative **7** with chiral amines (*R*)- and (*S*)- 2-phenylpropan-1-amine respectively according to GP-I.

(*R*) Deltazinone (**2k-*R***): **2k-*R*** was obtained with 90% *ee* (K_D = 12±4 nM). HPLC conditions: CHIRAPAK IC column, *i*PrOH / CH_2Cl_2 = 05/95, flow rate = 0.5 mL min^{-1}, major enantiomer: t_R = 98.0 min; minor enantiomer: t_R = 105.9 min.

(*S*) Deltazinone (**2k-*S***): **2k-*S*** was obtained with 93% *ee* (K_D = 5±3 nM). HPLC conditions: CHIRAPAK IC column, *i*PrOH / CH_2Cl_2 = 05/95, flow rate = 0.5 mL min^{-1}, major enantiomer: t_R = 104.8 min; minor enantiomer: t_R = 99.3 min.

^{1}H and ^{13}C spectra of compound **3**

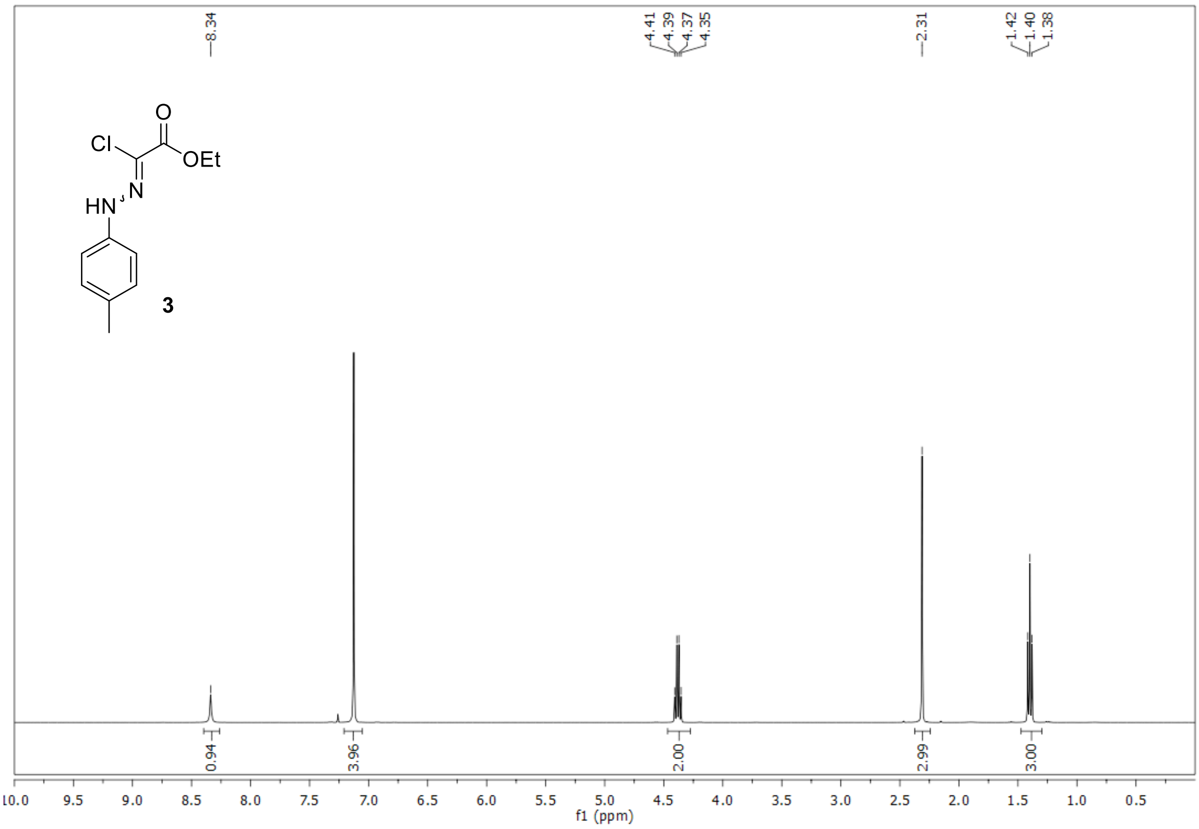

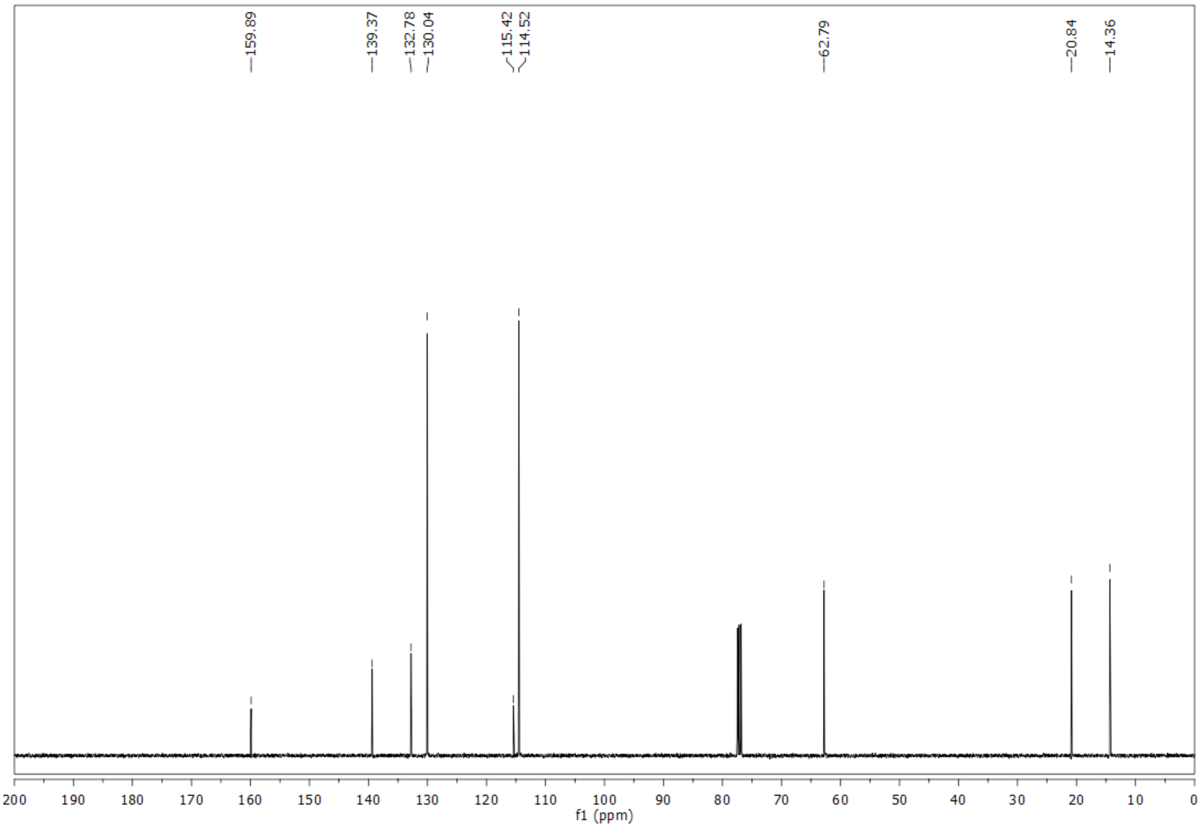

^{1}H and ^{13}C spectra of compound **5**

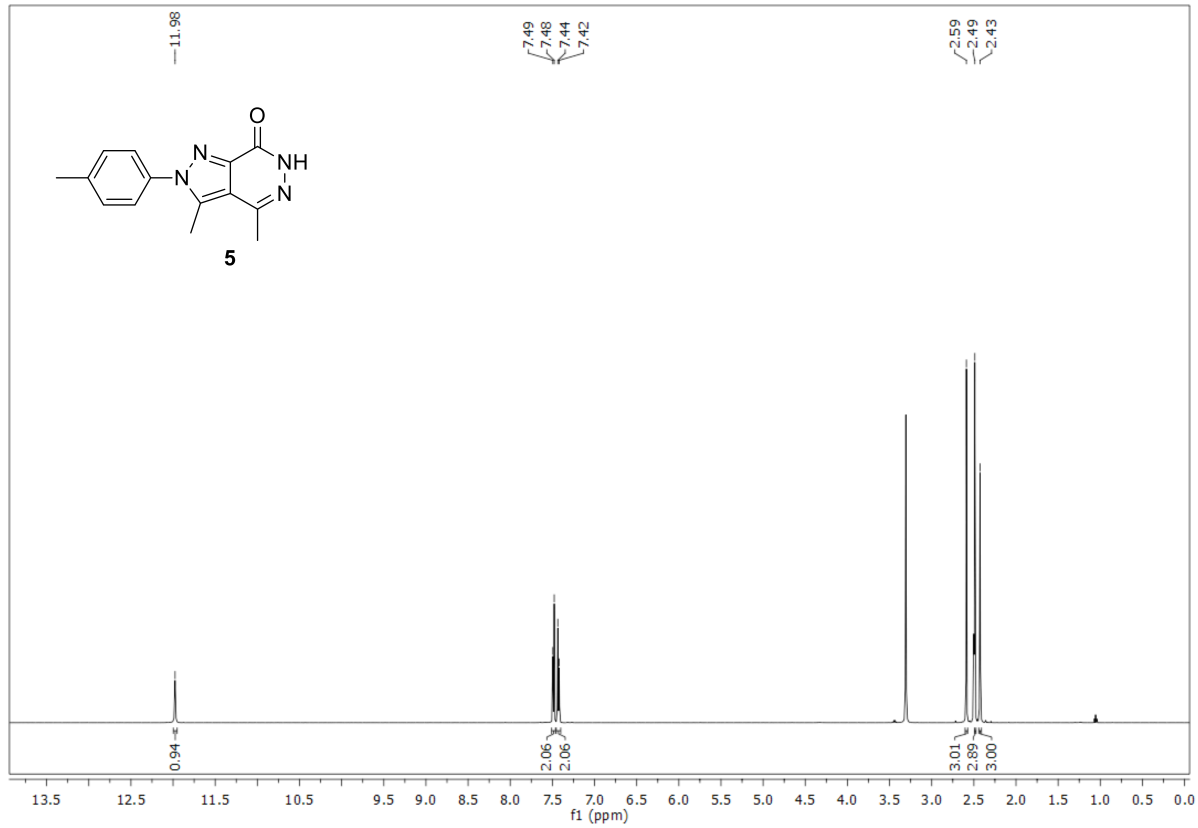

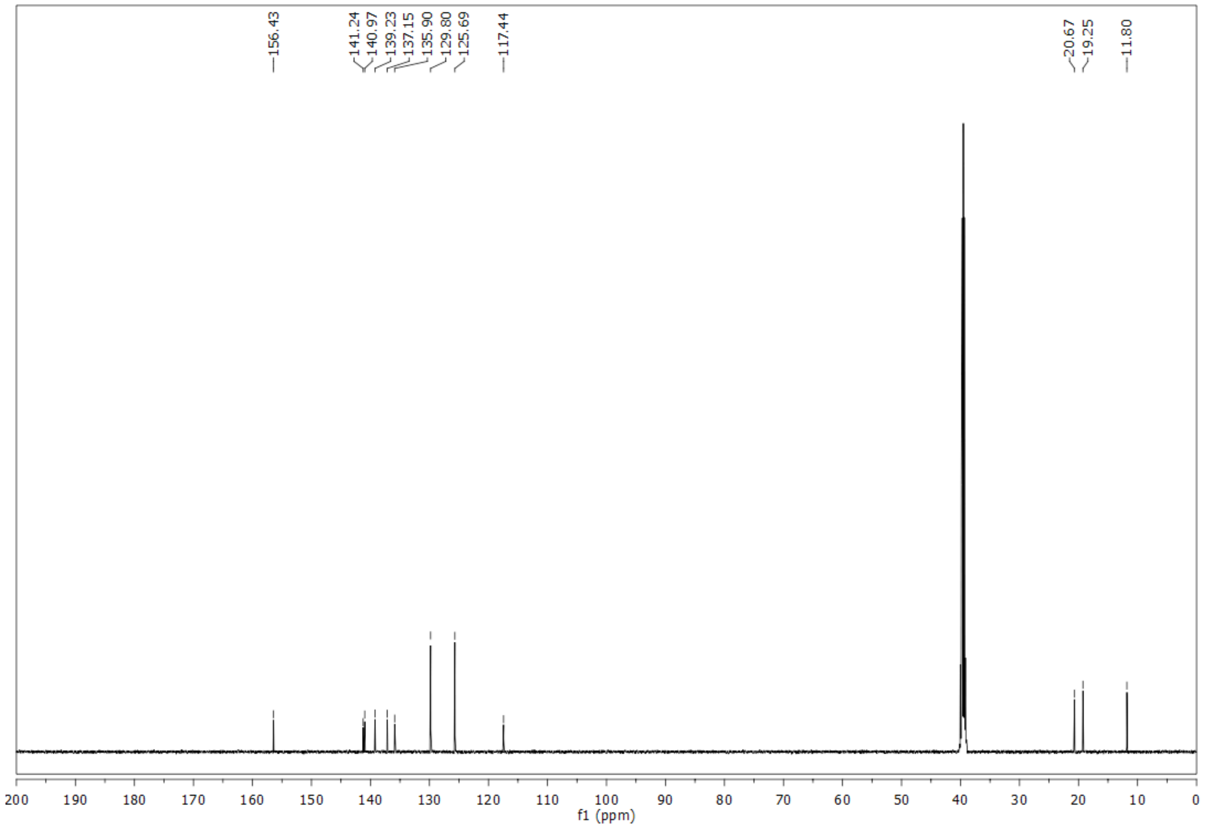

^{1}H and ^{13}C spectra of compound **6**

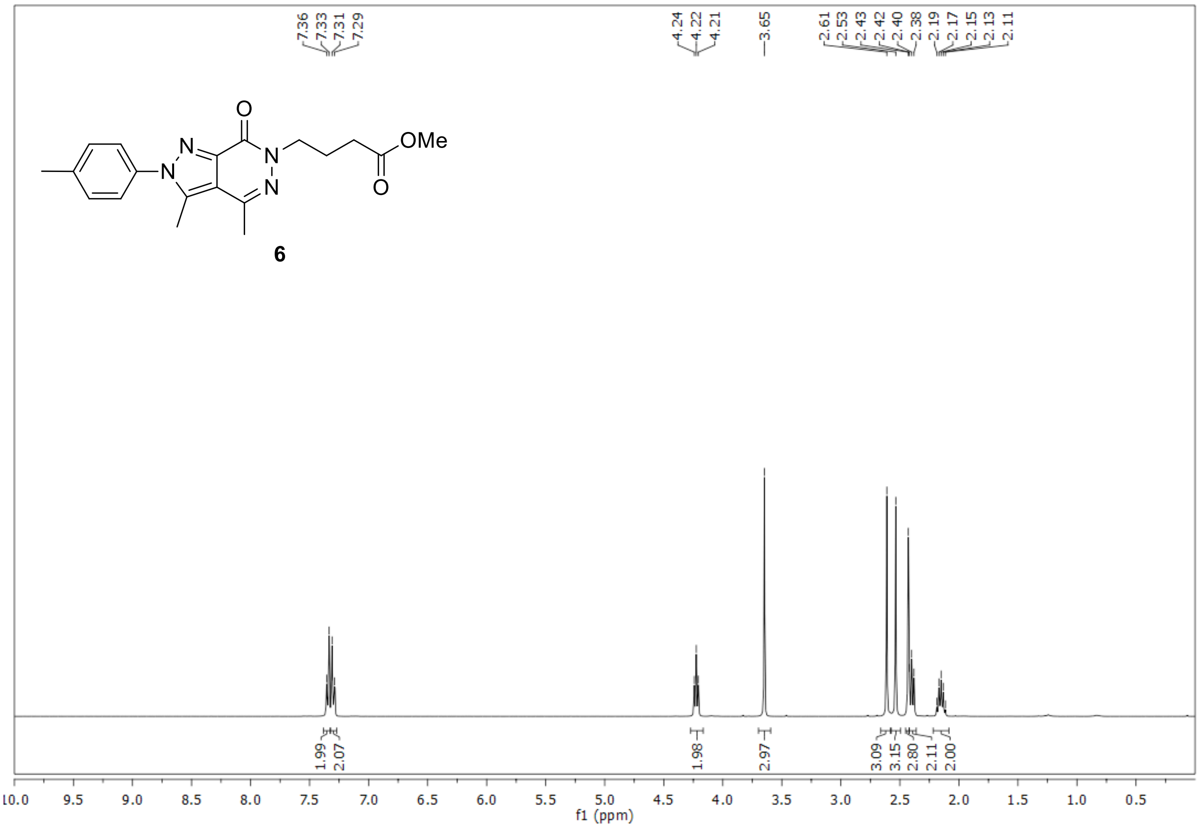

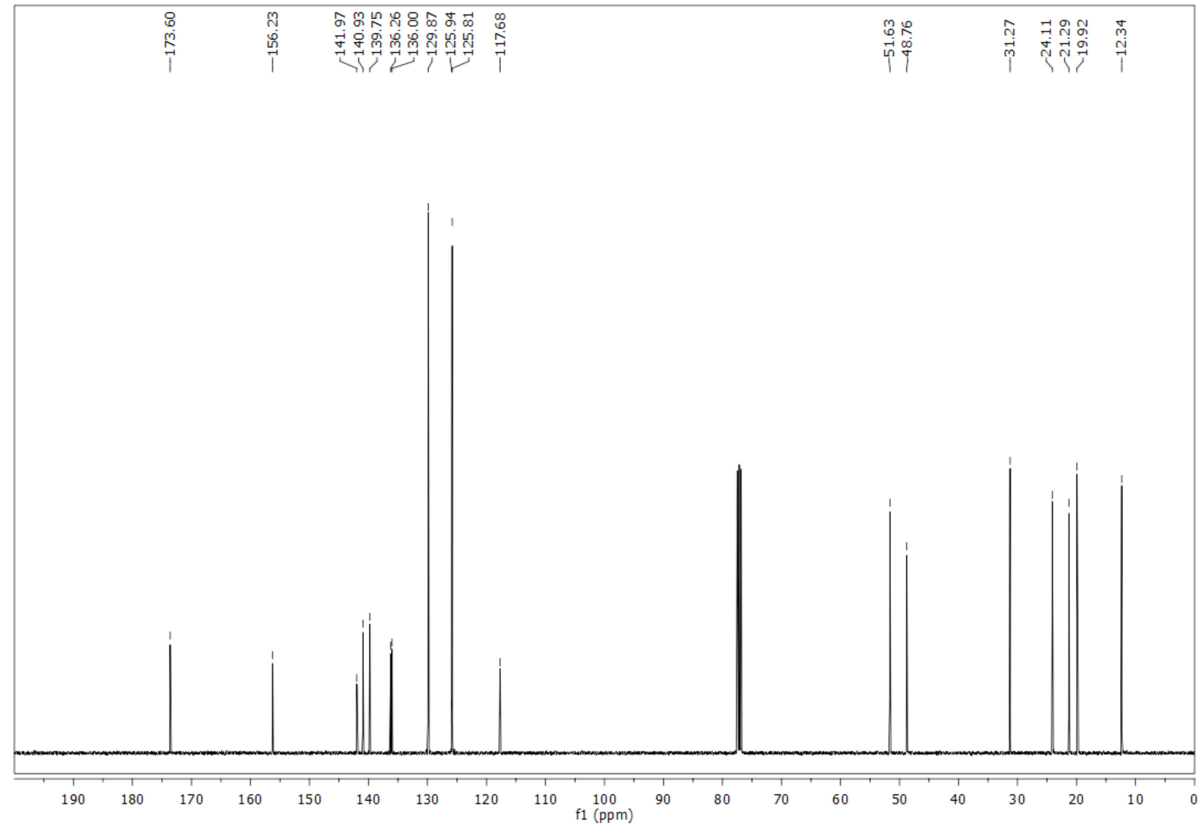

^{1}H and ^{13}C spectra of compound **7**

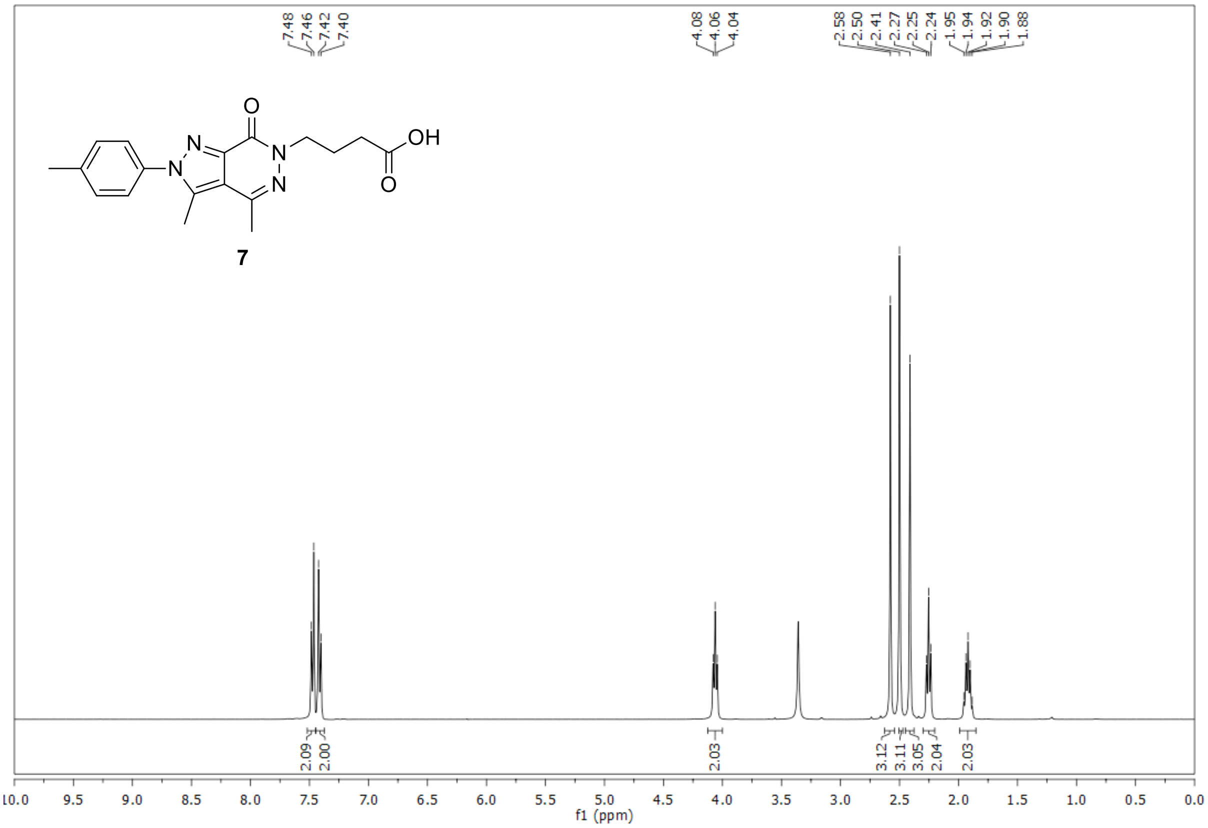

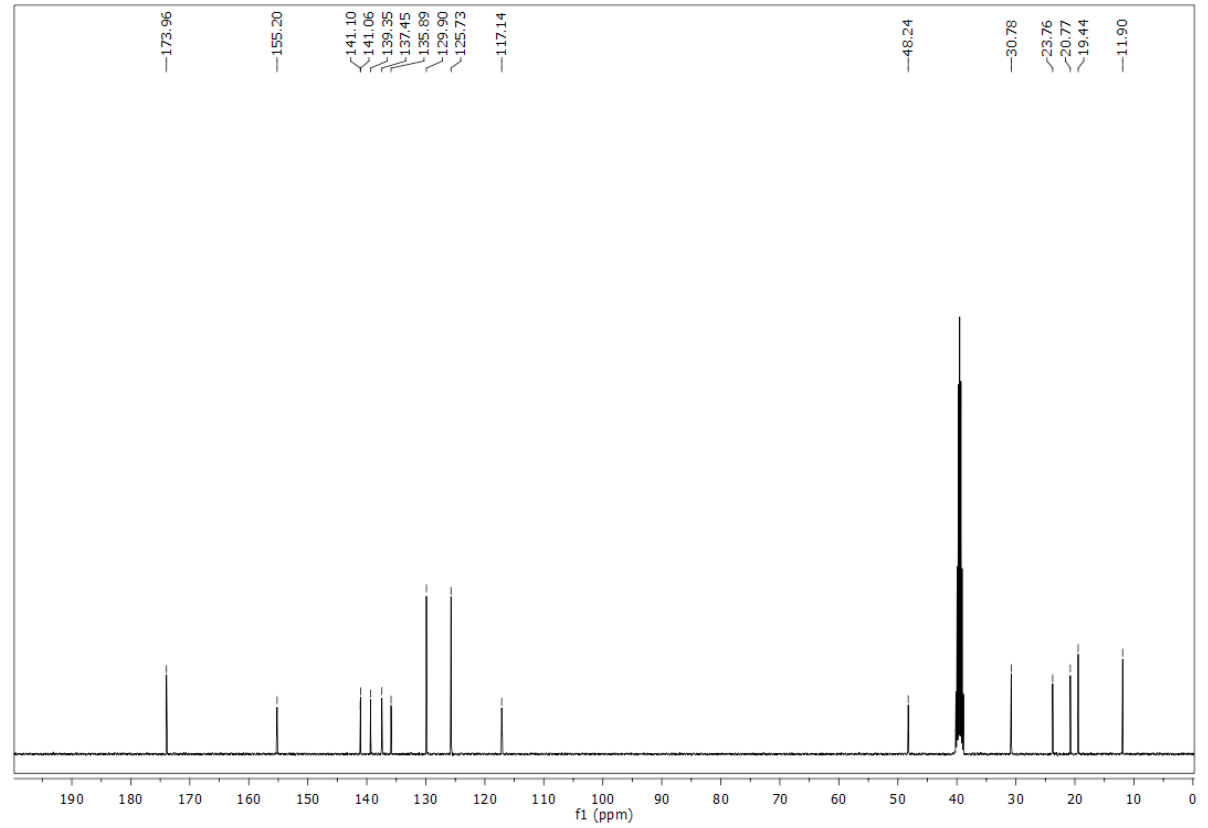

^{1}H and ^{13}C spectra of compound **2k**

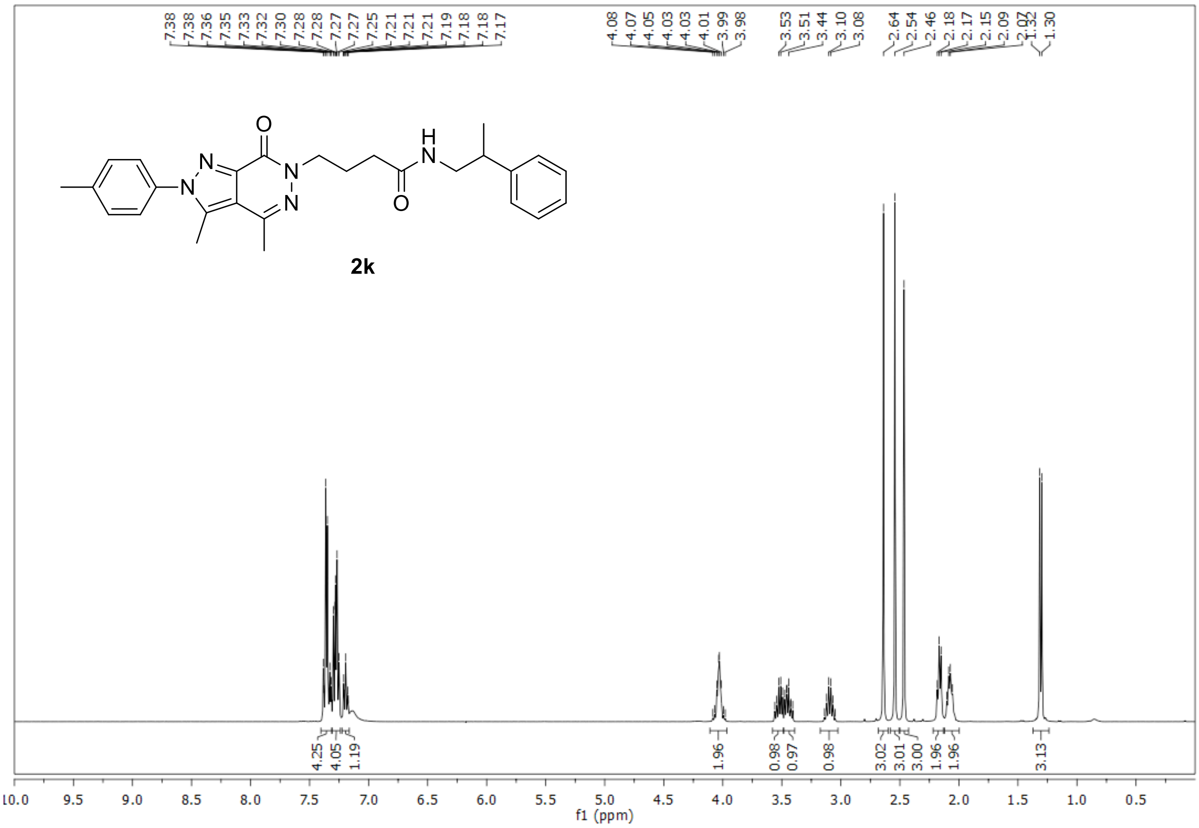

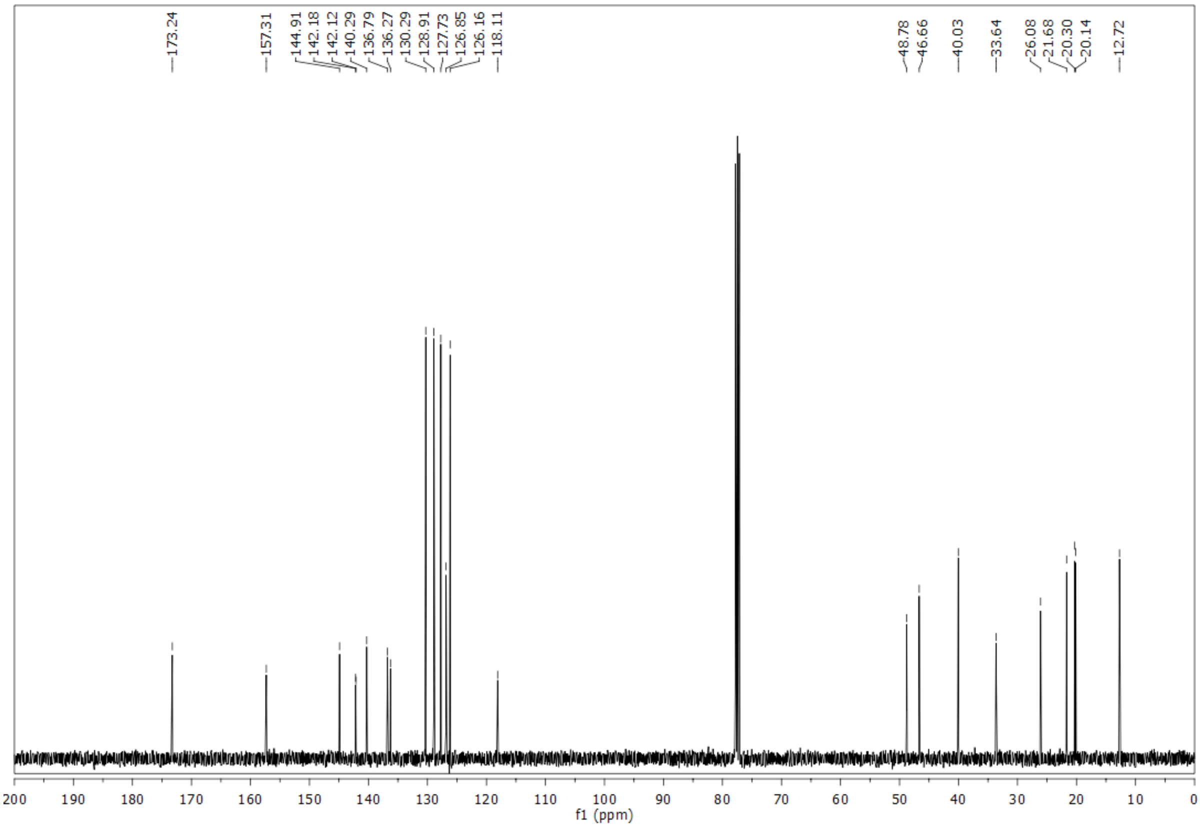

HPLC trace of (*R*)-deltazinone (**2k-*R***)

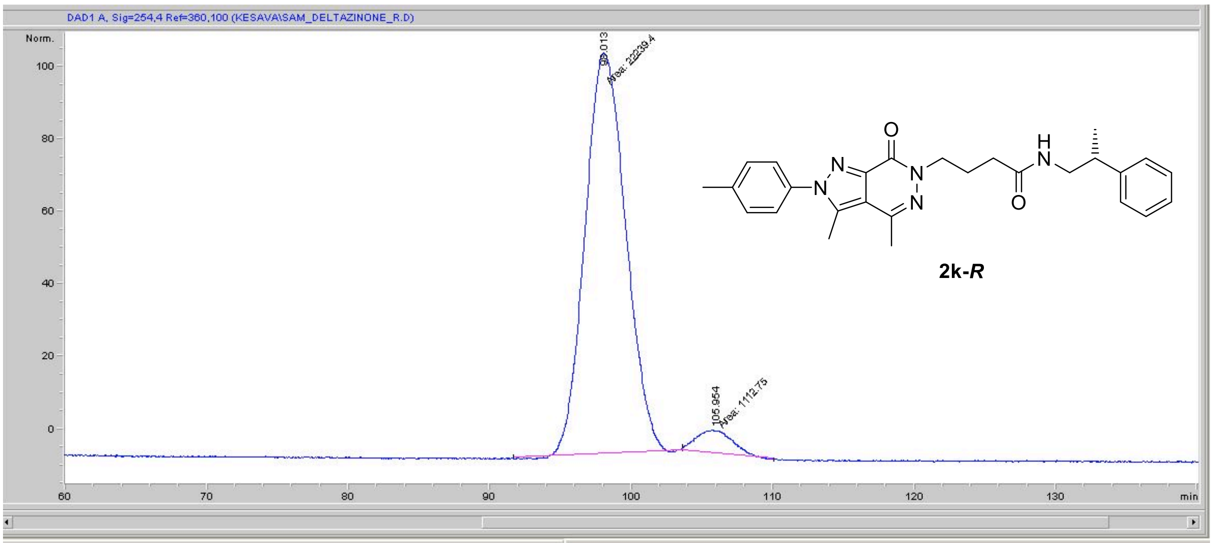

File Information	
LC-File	SAM_DELTAZINONE_R.D
File Path	C:\CHEM32\1\DATA\KESAVA\
Date	03-Oct-15, 14:35:31
Sample	
Sample Info	
Barcode	
Operator	Kesava
Method	HD_4RUN_95D5C.M
Analysis Time	149.993 min
Sampling Rate	0.0067 min (0.402 sec), 22500 datapoints

#	Time	Area	Height	Width	Area%	Symmetry
1	98.013	22239.4	110.9	3.3435	95.235	0.847
2	105.954	1112.7	6.2	2.9776	4.765	0.699

HPLC trace of (*S*)-deltazinone (**2k-*S***)

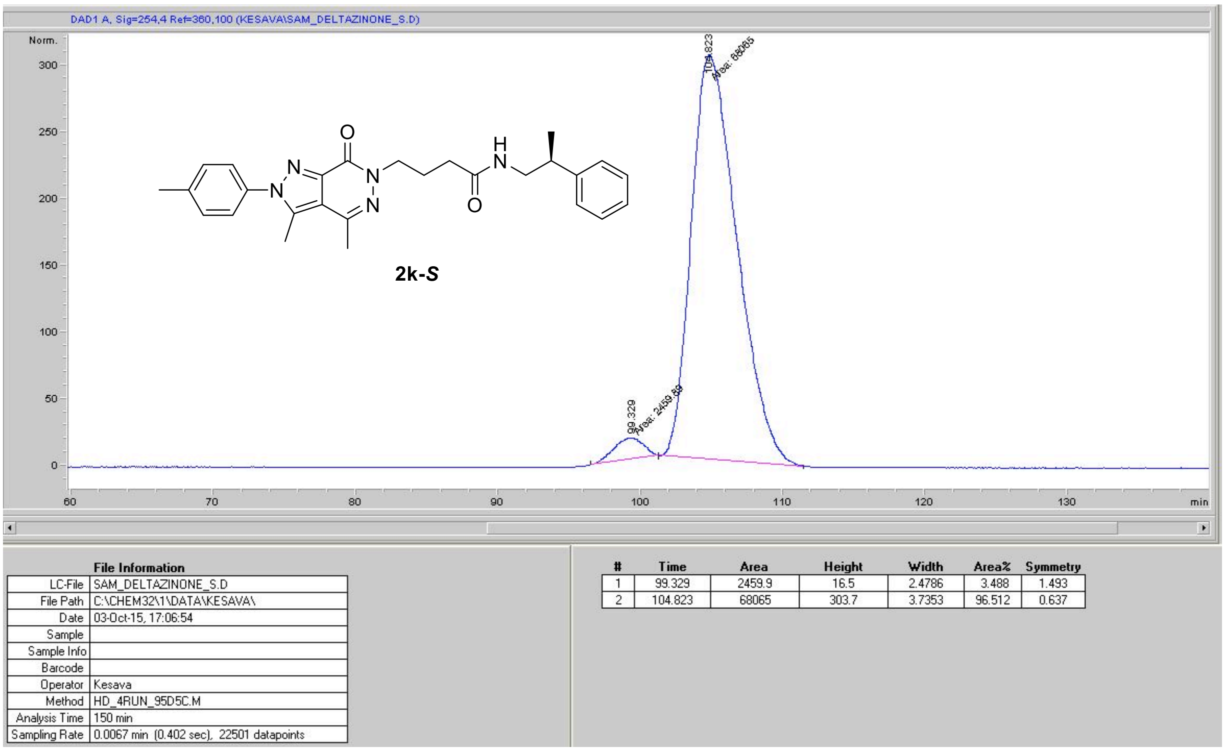

File Information	
LC-File	SAM_DELTAZINONE_S.D
File Path	C:\CHEM32\1\DATA\KESAVA\
Date	03-Oct-15, 17:06:54
Sample	
Sample Info	
Barcode	
Operator	Kesava
Method	HD_4RUN_95D5C.M
Analysis Time	150 min
Sampling Rate	0.0067 min (0.402 sec), 22501 datapoints

#	Time	Area	Height	Width	Area%	Symmetry
1	99.329	2459.9	16.5	2.4786	3.488	1.493
2	104.823	68065	303.7	3.7353	96.512	0.637

Supplementary References:

1. Zimmermann, G. *et al.* Small molecule inhibition of the KRAS-PDEdelta interaction impairs oncogenic KRAS signalling. *Nature* **497**, 638-642 (2013).
2. Zhang, Y., Huo, M., Zhou, J. & Xie, S. PKSolver: An add-in program for pharmacokinetic and pharmacodynamic data analysis in Microsoft Excel. *Computer methods and programs in biomedicine* **99**, 306-314 (2010).
3. Matiichuk, V.S., Potopnyk, M.A. & Obushak, N.D. Molecular Design of Pyrazolo[3,4-d]pyridazines. *Russ J Org Chem+* **44**, 1352-1361 (2008).
